# Optimisation of the coalescent hyperbolic embedding of complex networks

**DOI:** 10.1038/s41598-021-87333-5

**Published:** 2021-04-16

**Authors:** Bianka Kovács, Gergely Palla

**Affiliations:** 1 Department of Biological Physics, Eötvös Loránd University, Pázmány P. stny. 1/A, Budapest, 1117 Hungary; 2 MTA-ELTE Statistical and Biological Physics Research Group, Pázmány P. stny. 1/A, Budapest, 1117 Hungary; 3 Health Services Management Training Centre, Semmelweis University, Kútvölgyi út 2, Budapest, 1125 Hungary

**Keywords:** Complex networks, Statistical physics

## Abstract

Several observations indicate the existence of a latent hyperbolic space behind real networks that makes their structure very intuitive in the sense that the probability for a connection is decreasing with the hyperbolic distance between the nodes. A remarkable network model generating random graphs along this line is the popularity-similarity optimisation (PSO) model, offering a scale-free degree distribution, high clustering and the small-world property at the same time. These results provide a strong motivation for the development of hyperbolic embedding algorithms, that tackle the problem of finding the optimal hyperbolic coordinates of the nodes based on the network structure. A very promising recent approach for hyperbolic embedding is provided by the noncentered minimum curvilinear embedding (ncMCE) method, belonging to the family of coalescent embedding algorithms. This approach offers a high-quality embedding at a low running time. In the present work we propose a further optimisation of the angular coordinates in this framework that seems to reduce the logarithmic loss and increase the greedy routing score of the embedding compared to the original version, thereby adding an extra improvement to the quality of the inferred hyperbolic coordinates.

## Introduction

Network theory has become ubiquitous in the study of complex systems composed of many interacting units^1–3^. Over the last two decades, the overwhelming number of studies using this approach in systems ranging from metabolic interactions to the level of the global economy have shown that the statistical analysis of the underlying graph structure can highlight non-trivial properties and reveal previously unseen relations^1–5^. Probably the most important universal features of networks representing real systems are the small-world property^6,7^, the high clustering coefficient^8^ and the scale-free degree distribution^9,10^. On the modelling ground, a large number of network models were proposed for capturing one (or several) of these properties in a simple mathematical framework, and a quite notable example among these is provided by the PSO model^11^, which reproduces all three properties simultaneously in a natural manner. In this approach the nodes are placed one by one on the native disk representation^12^ of the 2D hyperbolic plane with a logarithmically increasing radial coordinate and a random angular coordinate, and links are drawn with probabilities determined by the hyperbolic distance between the node pairs. In vague terms, the degree of nodes is determined by their radial coordinate (lower distance from the origin corresponds to larger degree), and the angular proximity of the nodes can be interpreted as a sort of similarity, where more similar nodes have a higher probability to be connected.

The idea that hidden metric spaces can play an important role in the structure of complex networks first arose in a study focusing on the self-similarity of scale-free networks^13^. This was followed by reports showing the signs of hidden geometric spaces behind protein interaction networks^14,15^, the Internet^16–20^, brain networks^21,22^, or the world trade network^23^, also revealing important connections between the the navigability of networks and hyperbolic spaces^16,24,25^. In parallel, practical tools for generating hyperbolic networks^26^ and methods for measuring the hyperbolicity of networks were also proposed^27,28^. In the recent years the geometric nature of weights^29^ and clustering^30,31^ was revealed, and further variants of the original PSO model were proposed for generating random hyperbolic networks with communities^32,33^.

The very notable advancements in the hidden metric space related research provide a strong motivation for the development of hyperbolic embedding techniques^34–38^, following the pioneering work in Ref.^17^, tackling the problem of inferring plausible coordinates for the nodes based on the network structure. One of the first methods pointing in this direction was HyperMap^34^, which optimises a logarithmic loss function obtained from the assumption that the network was generated according to a generalised version of the PSO model (referred to as the E-PSO model). In contrast, in Ref.^35^ an embedding based on a non-linear dimension reduction of the Laplacian matrix was proposed. Along a similar line, a whole family of embedding algorithms were studied in Ref.^36^, using different pre-weighted matrices encapsulating the network structure and multiple unsupervised dimension reduction techniques from machine learning. In this framework, after the dimension reduction the nodes are organised on a circular or quasilinear manifold from which the angular coordinates in the 2D hyperbolic plane can be obtained in a simple manner, whereas the radial coordinates are inferred based on the node degrees. The rationale behind such an approach is that for networks that are actually generated in a hyperbolic manner, the angular order of the nodes is preserved along the obtained low dimensional manifold. This phenomenon is referred to as ’angular coalescence’, and thus these methods are called coalescent embedding algorithms^36^. A combination of the Laplacian embedding and the likelihood optimisation based on the E-PSO model was proposed in Ref.^37^, and in a recent work the approach named Mercator was introduced^38^, where the Laplacian embedding is incorporated with optimisation with respect to the so-called ${\mathbb {S}}^1/{\mathbb {H}}^2$ model^13^.

In the present paper we propose an embedding algorithm combining a coalescent approach with likelihood optimisation based on the E-PSO model. One of the best performing dimension reduction techniques in Ref.^36^ was corresponding to the non-centered minimum curvilinear embedding (ncMCE)^39^, which also provides the starting point of our method. However, after obtaining the initial node coordinates based on ncMCE, we also apply an angular optimisation of the coordinates using a logarithmic loss function originating from the E-PSO model. We test the proposed approach on both synthetic and real network data, and compare the results with the outcome of HyperMap, the original ncMCE coalescent embedding and Mercator in terms of the achieved logarithmic loss and the greedy routing score (which is a model-free quality measure of the embeddings).

## Preliminaries and algorithm description

In the following, we briefly describe the necessary preliminaries together with our angular optimisation algorithm. Since the optimisation uses a logarithmic loss function based on the E-PSO model, we begin with the outline of the PSO and E-PSO models. This is followed by the definition of the loss function and a short description of two state-of-the-art embedding methods, the HyperMap and the Mercator algorithms. Finally, we provide a summary of the coalescent embedding algorithm ncMCE and describe the proposed optimisation of the angular coordinates.

### The E-PSO model

The basic idea of the PSO model is to place nodes on the native disk representation of the hyperbolic plane with increasing radial coordinates and random angular coordinates, and connect the node pairs with a linking probability depending on their hyperbolic distances. The parameters of the model are the curvature of the hyperbolic plane $K<0$ parametrised by $\zeta = \sqrt{-K}$, the total number of nodes *N*, the average degree $< k>$ parametrised by $m=< k>/2$, the popularity fading parameter $\beta \in (0,1]$ controlling the outward drift of the nodes, and the ’temperature’ $T\in [0,1)$ regulating the average clustering coefficient of the generated network. Initially the network is empty, and the nodes are placed on the hyperbolic disk in an iterative manner according to the following rules:

1. At iteration *i* the new node *i* appears with the radial coordinate $r_{ii} = \frac{2}{\zeta }\ln i$ and a uniformly random angular coordinate $\theta _i\in [0,2\pi )$. (The double indexing of the radial coordinate is for a simple bookkeeping of the position during the outward drift specified in the next rule).
2. The radial coordinates of all previous nodes $j<i$ are increased as $r_{ji} = \beta r_{jj}+(1-\beta )r_{ii}$. (Thus, the first index of the node position refers to the moment of birth, whereas the second index corresponds to the actual time step). This repeated outward shift in the node positions is usually referred to as ’popularity fading’, since nodes closer to the origin of the hyperbolic disk are close (in the hyperbolic sense) to a higher number of other nodes compared to nodes on the periphery.
3. The new node *i* is attached to the already existing nodes as follows:
  - If the number of previous nodes is *m* or smaller, then *i* is connected to all of them.
  - Otherwise, if $T=0$, then node *i* is connected to the *m* closest nodes according to the hyperbolic distance $x_{ij}$. For nodes with polar coordinates $(r_{ii},\theta _i)$ and $(r_{ji},\theta _j)$ this can be calculated from the hyperbolic law of cosines as
    1 $$\begin{aligned} \cosh (\zeta x_{ij}) = \cosh (\zeta r_{ii})\cosh (\zeta r_{ji})-\sinh (\zeta r_{ii})\sinh (\zeta r_{ji})\cos (\Delta \theta ), \end{aligned}$$
    where the angular difference $\Delta \theta$ is given by $\Delta \theta =\pi -\left| \pi -\left| \theta _i -\theta _j\right| \right|$.
  - If $i>m+1$ and $T>0$, then node *i* is connected to nodes $j<i$ with a probability depending on the hyperbolic distance $x_{ij}$ as
    2 $$\begin{aligned} p(x_{ij})=\frac{1}{1+{\mathrm {e}}^{\frac{\zeta }{2T}(x_{ij}-R_i)}}, \end{aligned}$$
    where the cutoff distance $R_i$ is given by
    3 $$\begin{aligned} R_i = \left\{ \begin{array}{ll} r_{ii}-\frac{2}{\zeta }\ln \left( \frac{2T}{\sin (T\pi )}\cdot \frac{1-{\mathrm {e}}^{-\frac{\zeta }{2}(1-\beta )r_{ii}}}{m(1-\beta )}\right) &{} {\mathrm {if}}\;\; \beta < 1, \\ r_{ii} - \frac{2}{\zeta }\ln \left( \frac{T}{\sin (T\pi )}\cdot \frac{\zeta r_{ii}}{m}\right) &{} {\mathrm {if}} \;\; \beta =1. \end{array} \right. \end{aligned}$$
    The above choice of $R_i$ ensures that the expected number of realised connections from *i* to previous nodes is *m*.
    The networks generated according to these rules have the small-world property, are scale-free (with a degree decay exponent equal to $1+1/\beta$), and with an appropriate choice of *T* can be made also highly clustered (lower temperature results in larger average clustering coefficient)^11^. However, an important criticism raised against the PSO model is that for subgraphs spanning between nodes having a degree $k>k_{\mathrm{min}}$, we cannot observe the densification law seen in a couple of real networks when $k_{\mathrm{min}}$ is increased^38^.
    A generalisation of the PSO model circumventing this problem was proposed in Refs.^11,34^, where the iteration rules listed above are extended by adding extra links also between already existing nodes as follows:
4. For a randomly chosen, non-connected node pair $j,l<i$ draw a link with probability $p(x_{jl})$, where the hyperbolic distance $x_{jl}$ is calculated from the coordinates $(r_{ji},\theta _j)$ and $(r_{li},\theta _l)$, and $p(x_{jl})$ is evaluated according to equation (2). Repeat this until $L_{+}$ number of extra links are created.

The main effects of these so-called internal links are that the average degree of the generated network is modified to $< k>=2(m + L_{+})$, and the average internal degree of the subgraphs between nodes with degrees larger than a certain $k_{\mathrm{min}}$ becomes increasing as a function of $k_{\mathrm{min}}$. The expected number of links created between node *i* and all previous nodes by the end of the network growth (assuming altogether *N* nodes) can be given as^34^

4 $$\begin{aligned} {\bar{m}}_i = m + {\bar{L}}_i \simeq m + L_{+} \frac{2(1-\beta )}{(1-N^{-(1-\beta )})^2(2\beta -1)}\left[ \left( \frac{N}{i}\right) ^{2\beta -1}-1\right] \left( 1-i^{-(1-\beta )}\right) . \end{aligned}$$

An equivalent model with only external links (connections emerging always with the newly appearing node) was also formulated in Ref.^34^, which is referred to as the E-PSO model. In this approach we return to the iteration rules 1–3 of the original PSO model, and omit rule 4. from the generalised version. However, a very important difference compared to the original model is that the expected number of links connected to the newly appearing nodes is no longer constant, instead it changes during the iterations. In order to obtain on average the same number of links connected to any given node as in the generalised PSO with the internal links, the parameter *m* in step 3. is replaced by the expression given in Eq. (4).

To generalise the concept of internal links further, it is also conceivable that after a while some of the connections are deleted. Along this line, we can extend the generalised PSO model with the deletion of the link between $L_-$ number of connected pairs of old nodes at each time step. But how should the links be selected for deletion? If the temperature *T* is set to 0, when creating new (either external or internal) links, we have to always connect the node pair from the candidates that is characterised by the smallest hyperbolic node to node distance. The opposite of this deterministic connection rule is easy to phrase: for $T=0$ in each deletion step the link connecting the hyperbolically furthermost nodes is split up. Consequently, for $T=0$ the case $L_+=L_-$ gives back exactly the original PSO model.

At $T>0$, a natural extension of the above concept is to assume a link removal process where the probability that a link will not be deleted corresponds to the usual PSO linking probability, and the complementary probability of this is the removal probability, according to which we remove at each time step $L_-$ number of internal links at random. In this way, when $L_+=L_-$ in the generalised PSO model, we add and remove the same number of internal links at each time step, and therefore, the resulting networks become equivalent to the networks generated by the original PSO model.

By taking $L=L_+ - L_-$ as the net number of added and removed internal links per time step, we can also consider the analogous generalised E-PSO model, where all connections are created as external links at the appearance of the new nodes, without any additional link insertion or deletion. In this framework, by adjusting ${\bar{m}}_i$, the expected number of links connected to the new node *i* at its appearance, the resulting network can be made equivalent to the generalised PSO model with the insertion and the deletion of internal links. The method is straightforward, we can simply use

5 $$\begin{aligned} {\bar{m}}_i = m+{\bar{L}}_i \simeq m+L\cdot \frac{2(1-\beta )}{(1-N^{-(1-\beta )})^2(2\beta -1)}\left[ \left( \frac{N}{i}\right) ^{2\beta -1}-1\right] \left( 1-i^{-(1-\beta )}\right) , \end{aligned}$$

where the only (but important) difference compared to equation (4) is that *L* can also be negative, whereas $L_+$ in equation (4) is always non-negative.

**Figure 1:**
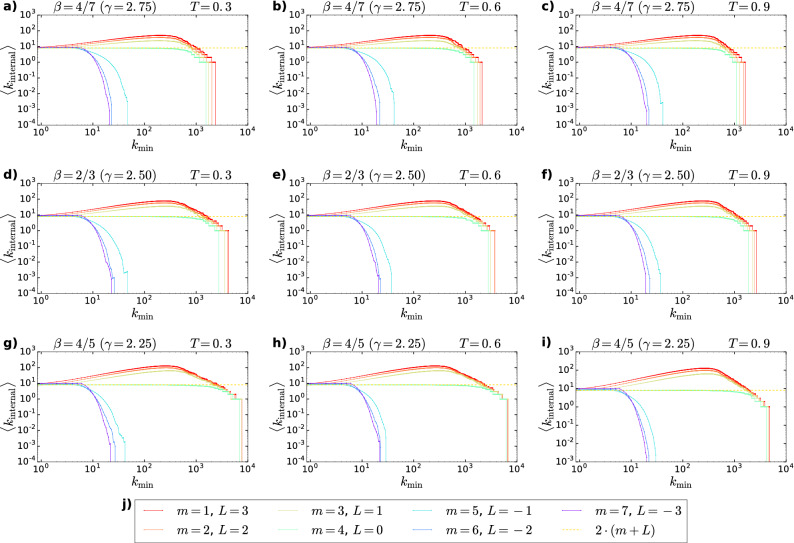
Average internal degree of subgraphs spanning between nodes with degrees larger than a threshold as a function of the degree threshold for synthetic networks generated by the E-PSO model using different parameters. With each parameter setting one network was generated with size $N=100,000$. The parameter $\zeta$ was always set to 1. Each panel corresponds to a certain $\beta -T$ setting given in the title of the subplot. The different colours of the curves indicate the different settings of the parameters *m* and *L*, as listed in panel j). The expected average degree $< k>=2\cdot (m+L)$ was 8 for each network.

In order to demonstrate that the introduction of the internal links during the network generation process can solve the problem of the lack of the densification in the subgraphs between nodes having a degree $k>k_{\mathrm{min}}$ observed in the original PSO model, in Fig. 1 we plot the average internal degree $< k_{\mathrm {internal}}>$ of the subgraphs spanning between nodes having a degree larger than a certain threshold $k_{\mathrm{min}}$ as a function of $k_{\mathrm{min}}$ for both positive and negative *L* values (indicated by different colours) at different $\beta$ and *T* parameters. When *L* is positive (analogous to generalised PSO networks, where at each time step the number of newly created internal links is larger than the number of deleted internal links), the average internal degree becomes larger as the degree threshold begins to increase. For $L=0$ (corresponding to the case of the original PSO model) the average internal degree remains constant until the degree threshold does not become so large that the subgraphs become extremely small. And finally, for negative *L* (analogous to generalised PSO networks, where at each time step more internal links are deleted than created) with the increase of the degree threshold the average internal degree decreases even for relatively small values of the threshold. Note that the shape of the $< k_{\mathrm {internal}}>-k_{\mathrm{min}}$ curve does not depend on the popularity fading parameter $\beta$, and thus, neither on the exponent $\gamma$ of the degree distribution, as opposed to the ${\mathbb {S}}^1/{\mathbb {H}}^2$ model^13^, where the average internal degree is an increasing function of the degree threshold only for $\gamma <3$.

### Calculating the logarithmic loss

Assuming a network obtained from the E-PSO model, the probability for observing a connection between nodes having the final coordinates $(r_{iN},\theta _i)$ and $(r_{jN},\theta _j)$ at the end of the network generation process was also given in Ref.^34^ in the form of

6 $$\begin{aligned} {\tilde{p}}(x_{ij}) = \frac{1}{N-i_{\mathrm{min}}+1}\sum _{i=i_{\mathrm{min}}}^N\frac{1}{1+{\mathrm {e}}^{\frac{\zeta }{2T}(x_{ij}-R_N+\Delta _i)}}\simeq \frac{1}{1+{\mathrm {e}}^{\frac{\zeta }{2T}(x_{ij}-R_N)}}, \end{aligned}$$

where $x_{ij}$ stands for the hyperbolic distance calculated based on Eq. (1), $i_{\mathrm{min}}=\max (2,\lceil N{\mathrm {e}}^{-\frac{\zeta x_{ij}}{4(1-\beta )}}\rceil )$, $R_N$ is given by equation (3), and $\Delta _i=\frac{2}{\zeta }\ln \left[ \left( \frac{N}{i}\right) ^{2\beta -1}\frac{mI_i}{m_iI_N}\right]$ with $I_i=\frac{1}{1-\beta }(1-i^{-(1-\beta )})$. Using equation (6), the likelihood of observing an adjacency matrix $A_{ij}$ for given final hyperbolic distances $x_{ij}$ can be calculated from

7 $$\begin{aligned} {\mathcal {L}}_{A} \equiv {\mathcal {L}}(A_{ij}\mid \{ r_{iN},\theta _i \}, m, L,\zeta ,\beta ,T)=\prod _{1\le j <i\le N} {\tilde{p}}(x_{ij})^{A_{ij}}\left[ 1-{\tilde{p}}(x_{ij})\right] ^{1-A_{ij}}. \end{aligned}$$

However, when we are interested in the goodness of the fit for an embedding, we need the conditional probability of the node coordinates given the adjacency matrix and the model parameters, which according to Bayes’ rule can be expressed as

8 $$\begin{aligned} {\mathcal {L}}_{r,\theta }\equiv {\mathcal {L}}_{r,\theta }(\{ r_{iN},\theta _i \} \mid A_{ij} , m, L,\zeta ,\beta ,T)= \frac{{\mathcal {L}}(\{ r_{iN},\theta _i \} \mid m, L,\zeta ,\beta ,T)\cdot {\mathcal {L}}_{A}}{{\mathcal {L}}(A_{ij}\mid m, L,\zeta ,\beta ,T)}, \end{aligned}$$

where ${\mathcal {L}}(\{ r_{iN},\theta _i \} \mid m, L,\zeta ,\beta ,T)$ corresponds to the conditional probability for obtaining the final node coordinates $\{ r_{iN},\theta _i \}$ given the model parameters, and ${\mathcal {L}}(A_{ij}\mid m, L,\zeta ,\beta ,T)$ is the conditional probability for receiving the adjacency matrix $A_{ij}$ given the model parameters. Since the angular coordinates are uniformly random and the radial coordinates (according to the iteration rules 1–2) depend only on $\zeta$ and $\beta$, it can be shown that^34^

9 $$\begin{aligned} {\mathcal {L}}(\{ r_{iN},\theta _i \} \mid m, L,\zeta ,\beta ,T)= {\mathcal {L}}(\{ r_{iN},\theta _i \} \mid \zeta ,\beta ) = \frac{1}{(2\pi )^N}\prod _{i=1}^N\frac{\zeta }{2\beta }{\mathrm {e}}^{\frac{\zeta }{2\beta }(r_{iN}-r_{NN})}, \end{aligned}$$

where $$r_{NN}=\frac{2}{\zeta }\ln N$$.

If we are given an input network together with model parameters, the maximum likelihood estimate for the node coordinates is formally that set $\{ r_{iN}^*,\theta _i^* \}$ for which ${\mathcal {L}}_{r,\theta }$ is maximal. As usual, technically it is far more convenient to maximise the logarithm of ${\mathcal {L}}_{r,\theta }$, which is equivalent to minimising $-\ln {\mathcal {L}}_{r,\theta }$ given by

10 $$\begin{aligned} -\ln {\mathcal {L}}_{r,\theta } = C - \frac{\zeta }{2\beta }\sum _{i=1}^N r_{iN}-\sum _{i=1}^{N-1}\sum _{j=i+1}^N A_{ij}\ln {\tilde{p}}(x_{ij})-\sum _{i=1}^{N-1}\sum _{j=i+1}^N(1-A_{ij})\ln \left[ 1- {\tilde{p}}(x_{ij})\right] , \end{aligned}$$

where *C* is a constant independent from $\{ r_{iN},\theta _i \}$. The analytic solution for the optimal radial coordinates can be given as^34^

11a $$\begin{aligned} r_{ii}^*&=\frac{2}{\zeta }\ln i^*, \end{aligned}$$

11b $$\begin{aligned} r_{iN}^*&= \beta r_{ii}^* +(1-\beta )r_{NN}^*, \end{aligned}$$

where the optimal ordering of the nodes given by $i^*$ is following the node degrees, with the largest degree node in the network obtaining $i^*=1$, second largest degree node receiving $i^*=2$, etc., and Eq. (11a) corresponds to the initial radial coordinate of node $i^*$, whereas Eq. (11b) takes into account also the outward drift due to the popularity fading. The optimal solution for the angular coordinates cannot be expressed analytically in closed form, opening up the room for heuristic optimisation algorithms. After substituting in Eq. (10) the sum of the $r_{iN}^*$ values expressed from Eqs. (11a–b) as a function of the model parameters $\zeta$, *N* and $\beta$, the node arrangement dependent part of the negative log-likelihood can be written as

12 $$\begin{aligned} LL \equiv -\ln {\mathcal {L}}_{A} = -\sum _{i=1}^{N-1}\sum _{j=i+1}^N A_{ij}\ln {\tilde{p}}(x_{ij})-\sum _{i=1}^{N-1}\sum _{j=i+1}^N(1-A_{ij})\ln \left[ 1- {\tilde{p}}(x_{ij})\right] , \end{aligned}$$

which we shall refer to as the logarithmic loss from here on.

### Embedding with HyperMap

Probably the most well-known method for minimising the logarithmic loss is HyperMap, introduced in Ref.^34^ for embedding networks based on the E-PSO model. In this approach the nodes of the network are sorted and indexed in decreasing order of their degree. The node with the largest degree (indexed by $i=1$) is placed at the centre of the hyperbolic disk, and the rest of the nodes are introduced one by one, obtaining initial radial coordinates given by Eq. (11a). At the introduction of a new node, the radial coordinates of the previous nodes are updated according to the concept of popularity fading, and the angular coordinate of the new node is chosen by minimising a local version of the logarithmic loss, where contributions only from the already introduced nodes (including the new node) are taken into account. In an improved version of this approach further periodic correction steps are also applied for better adjustment of the angular coordinates. In our studies we have used this algorithm based on the code available from Ref.^34^, where further details of the method are also given.

### Embedding with Mercator

An alternative, very successful approach to embed networks into the hyperbolic space is offered by Mercator^38^. This method is based on adapting the Laplacian Eigenmaps approach^35,37^ to the ${\mathbb {S}}^1$ model^13^ in order to compute initial angular positions for the nodes. Once these are estimated, Mercator is optimising the angular coordinates further using the likelihood in the static ${\mathbb {S}}^1/{\mathbb {H}}^2$ model^38^. During this process, several possible new angular coordinates are examined for each node in the network, always keeping the one with the highest log-likelihood. The proposed new coordinates are always drawn from a Gaussian distribution centred around the mean angle of the neighbours of the given node, with a standard deviation set by half of the largest angular distance between the node and any of its neighbours. The computational complexity of this embedding method is ${\mathcal {O}}(N^2)$ for sparse networks, and its high performance in terms of the quality of the embedding was demonstrated on both synthetic networks generated by the PSO model and on real networks in Ref.^38^.

### Coalescent embedding with ncMCE

The short outline of the coalescent embedding methods is the following: first a weighted adjacency matrix is prepared (this step can be referred to as pre-weighting), based on which the node similarity matrix ${\mathbf {D}}$ is obtained, and then the angular coordinates of the nodes are gained by applying a dimension reduction technique to ${\mathbf {D}}$^36^. The rationale behind this approach is that when applied to a network that is known to be hyperbolic, a common node aggregation pattern can be observed in the embedding space which is circularly or linearly ordered (angular coalescence) according to the original angular coordinates in the hyperbolic space. An extensive study of different similarity matrices and dimension reduction methods was carried out in Ref.^36^, and according to tests on real input networks, the best greedy routing scores could be achieved by combining repulsion-attraction (RA) pre-weighting with ncMCE dimension reduction.

In this approach we first prepare a weighted adjacency matrix ${\mathbf {W}}$ with elements

13 $$\begin{aligned} W_{ij} = \frac{k_i+k_j+k_ik_j}{1+CN_{ij}}, \end{aligned}$$

where $k_i$ and $k_j$ denote the degree of nodes *i* and *j*, and $CN_{ij}$ stands for the number of common neighbors of these two nodes. The appearance of $CN_{ij}$ in the denominator of equation (13) provides a sort of ’repulsion’ between nodes having neighbours not in common (large $k_i$ and $k_j$ compared to $CN_{ij}$), resulting in larger $W_{ij}$ values, which reflect less similarity. Next the minimum weight spanning tree of the induced weighted network is prepared, and the entries of the similarity matrix ${\mathbf {D}}$ are given by the distance of the corresponding node pair in the spanning tree. The matrix element $D_{ij}$ can be interpreted as an estimate for the minimum curvilinear distance between node *i* and node *j*^39,40^.

The dimension reduction is carried out via singular value decomposition, corresponding to a factorisation of ${\mathbf {D}}$ as ${\mathbf {D}}={\mathbf {U}}{\varvec{\Sigma }}{\mathbf {V}}^T$, where ${\varvec{\Sigma }}$ is a diagonal matrix containing the singular values, from which we keep only the two largest ones (and put the rest to zero) in the following. Given a distance matrix, multidimensional scaling (MDS) can be used to determine that set of node coordinates which gives back all the pairwise distances. In our case the angular coordinates of the nodes are obtained from the matrix ${\mathbf {X}} = \left( \sqrt{{\varvec{\Sigma }}}\cdot {\mathbf {V}}^T\right) ^T$. Although already the coordinates in the second column of ${\mathbf {X}}$ could be regarded as the angular coordinates when re-scaled into the interval $[0,2\pi )$, according to Ref.^36^ we can obtain better results by applying an equidistant adjustment. Technically this is equivalent to distributing the angular coordinates in a regular uniform fashion over the interval $[0,2\pi )$, following the node order dictated by the second column of ${\mathbf {X}}$. The radial coordinates are obtained in the same way as in the logarithmic loss optimising methods, making use of Eqs. (11a–11b), where the radial order of the nodes is adjusted according to their degree.

### Optimisation of the angular coordinates

As mentioned in the Introduction, our embedding method combines the coalescent embedding approach with an optimisation of the angular coordinates based on the assumption that the network to be embedded was generated according to the E-PSO model. In the first state of the embedding process we apply the RA pre-weighting given by Eq. (13) for preparing the similarity matrix ${\mathbf {D}}$, and use the ncMCE dimension reduction technique described in the previous section to obtain the coordinate matrix ${\mathbf {X}}$. The initial angular coordinates inputted to our optimising algorithm correspond to the elements in the second column of ${\mathbf {X}}$ after equidistant adjustment.

During the optimisation we iterate over the network nodes according to their radial order (beginning with the innermost node), and examine in each iteration a *q* number of new angular positions for the current node, which are placed equidistantly between the second neighbours of the node according to the (current) angular node order. For each examined new position the logarithmic loss given by equation (12) is calculated, and if lower values are observed compared to the original one, the angular coordinate of the current node is updated to the best new position. The reason for limiting the arc of possible new positions between the two second neighbours is that the original coordinates obtained with the ncMCE method are usually already quite good; thus, only minor adjustments are needed for improving the embedding. Nevertheless, with this choice of boundaries we also allow swaps in the angular order of the nodes. (Whenever we have to update the angular coordinate of the current node to a new position between the first and second neighbours, the angular order is changed). By increasing the *q* number of new angular positions examined per iteration we also increase the chance for finding better node positions; however, since the computational cost of the method is proportional to *q*, on the other hand keeping *q* at low values pays off in terms of the running time. According to our experience, $q=6$ corresponds to a good compromise between these two options, allowing usually a fast improvement in *LL* at the beginning of the optimisation, and thus, we kept $q=6$ constant during all experiments shown in the paper.

Let us denote one iteration over all nodes as described above as a swapping round (due to the possibility of swaps in the angular order). After a few of these swapping rounds, in order to enable the settling of the angular positions to the true optimum allowed by the current angular order, we carry out a couple of non-swapping rounds of updates, where the *q* number of possible new angular positions are distributed only between the first angular neighbours of the current node. (e.g., in our experiments on synthetic networks, we used 5 swapping rounds followed by 3 non-swapping rounds.) The total number of rounds *n* can be either preset, or applying a stop condition based on the relative improvement in *LL* over the consecutive rounds is also a simple option.

In terms of complexity, the calculation of the change in the logarithmic loss *LL* when trying out a new node position involves the evaluation of $N-1$ number of terms, consequently the total number of calculation steps needed to perform the angular optimisation is proportional to $n\cdot N\cdot q\cdot (N-1)$ (where *n* denotes the aggregated number of swapping and non-swapping rounds). This means that the running time of our algorithm is linear in terms of *n* and *q*, and quadratic in terms of *N*. Thus, by keeping the number of optimisation rounds and the number of test positions per node low compared to the network size *N*, the computational complexity of the proposed embedding optimisation method is ${\mathcal {O}}(N^2)$, similarly to that of the original coalescent embedding approach based on ncMCE dimension reduction^36^.

Before actually showing the results of our algorithm, it is important to specify how we choose the parameters $\zeta ,\,m,\,L,\,\beta$ and *T* of the logarithmic loss, that are considered to be fixed during the angular optimisation. Following the standard practice in the literature, $\zeta$ (characterising the curvature of the hyperbolic plane) is always assumed to be 1. For $m,L,\beta$ and *T* we already mentioned in the description of the PSO and E-PSO models that these parameters are connected to the different statistical features of the generated graphs in mostly simple forms (e.g., the average degree is $< k>=2(m+L)$, etc.); thus, a reasonable estimate for these can be made by observing the corresponding properties of the network to be embedded. Nevertheless, for example fitting a power-law to the degree distribution of a network (in order to estimate the value of $\beta$) can be problematic in many cases, partly because of the uncertainty in the identification of the range of the degree distribution over that the power-law behaviour holds, and partly because the fitting itself can be complicated due to the occurrence of large fluctuations and the fact that the tail of the distribution usually falls into the regime of rare events. Therefore, instead of following the usual, frequently laborious procedures for estimating the embedding parameters, we applied a less burdensome method that is able to determine all the necessary parameters simultaneously.

**Figure 2:**
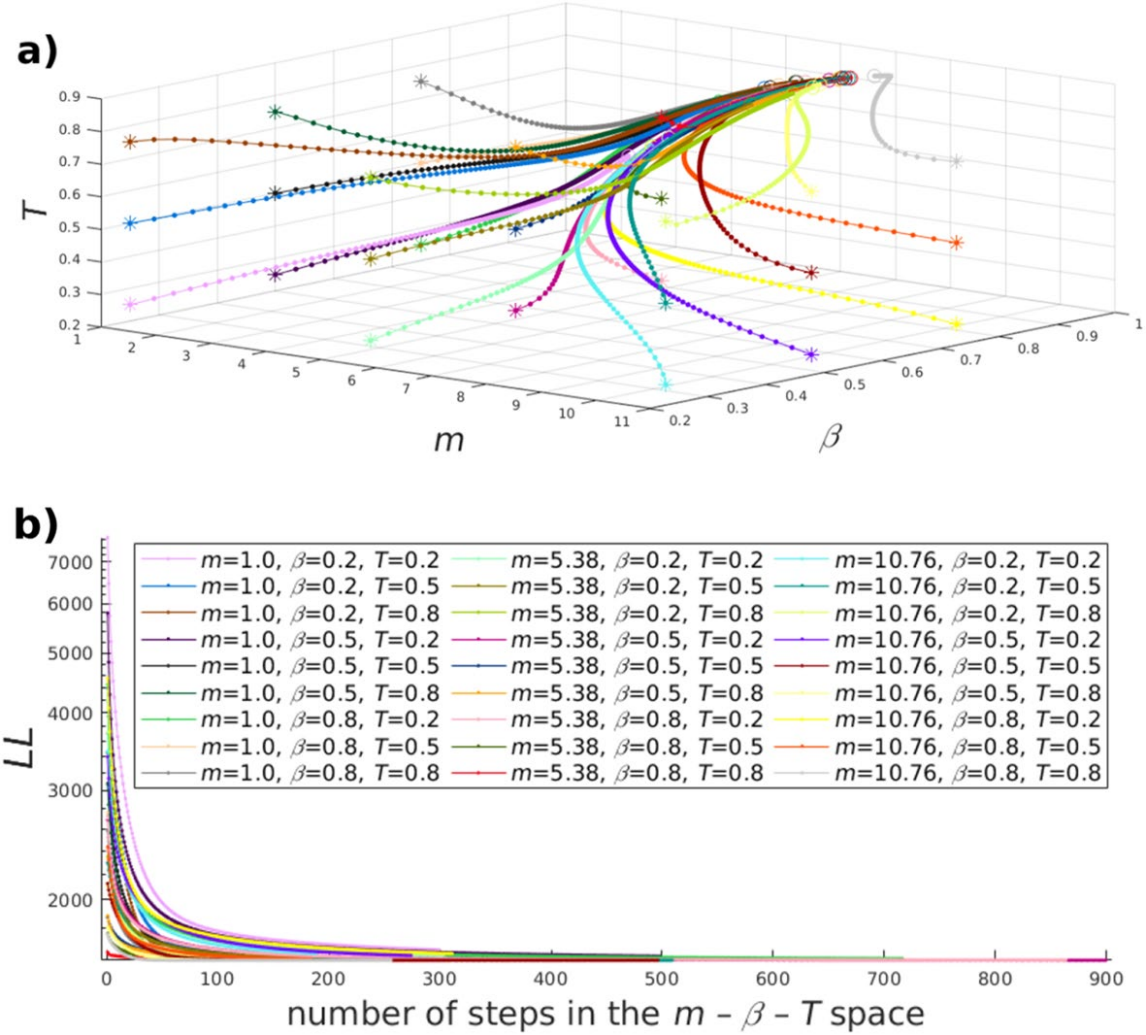
Illustration of the search for the optimal embedding parameters in the case of the Cambrian food web from the Burgess Shale. (**a**) Examples of the trajectories emerging during the gradient descent used for minimising the logarithmic loss *LL* of an ncMCE embedding in the case of different starting points in the $m-\beta -T$ parameter space. The termination points (indicated by circles) lie close to each other for all of the tested starting points (marked with stars), suggesting that a global optimum of the logarithmic loss does exist. (**b**) The logarithmic loss *LL* as a function of the number of steps taken along the trajectories starting from different points of the $m-\beta -T$ space shown in panel (**a**).

An important note is that when a network is assumed to be generated by the E-PSO model, the above-mentioned parameters characterise the adjacency matrix itself, and not a certain hyperbolic arrangement of the network. Therefore, we can assume that by optimising the parameters *m*, *L*, $\beta$ and *T* for just one particular embedding of the examined network, we can actually get close to a parametrisation of the E-PSO model that is congruent with the network in general. According to that, as a first step, we apply the ncMCE based coalescent embedding to obtain some initial coordinates for the nodes. Using these, we optimise $m,\,\beta$ and *T* simultaneously by minimising the logarithmic loss in Eq. (12) via a simple gradient descent in the $m-\beta -T$ parameter space, meaning that we take ever smaller steps in the direction of the negative gradient of *LL* (i.e. the vector $(-\frac{\partial LL}{\partial m},-\frac{\partial LL}{\partial \beta },-\frac{\partial LL}{\partial T})$) until we get so close to the optimum that the resultant step size becomes smaller than a given value, or in other words, until the optimum is approached with a given precision. During this procedure the node coordinates are kept fixed, and the value of *L* is calculated then from the relation $< k>=2(m+L)$; hence, it is actually not regarded as a free parameter on its own. As an illustration of the search in the parameter space, in Fig. 2a we show trajectories followed by our algorithm in the case of the Cambrian food web from the Burgess Shale^41^ (details about the studied networks are given in the Results section), and in Fig. 2b we display how the logarithmic loss *LL* improves when moving along these trajectories.

A remaining question is where to start the gradient descent from. In the case of embedding synthetic networks obtained from the PSO model, we started the search in the parameter space from the parameters used for the network generation, while for real networks we used $\beta =0.5$, $T=0.5$ and $m=< k>/2$ as the starting point. In accordance with our generalised E-PSO model, *L* was allowed to take negative values as well, i.e. *m* was allowed to take values larger than $< k>/2$. We permitted *m* to change between 1 and $2\cdot < k>$ (*m* never increased over this value even if it was not prohibited), while the value of $\beta$ and *T* was restricted to the interval [0.1, 0.99]. If the endpoint of a step would have fallen outside from the designated range of any of the parameters, we set the involved parameters to their allowed extremum in this step. The step size was tuned separately in each parameter’s direction. The size of the first step was set to the distance of the starting point from the permitted extremum falling in the direction of the initial negative derivative of the logarithmic loss multiplied by a constant factor smaller than 1, where the multiplying constant was the same for all the three partial derivatives and it was set experimentally to a value at which the algorithm seemed to eventually converge. The size of the following steps was calculated in each direction as the corresponding partial derivative multiplied by another constant factor; thus, together with the partial derivatives, the step sizes declined as the optimum was approached. The multiplying constant used from the second step was calculated by dividing the size of the first step in the given direction by the absolute value of the corresponding initial partial derivative. This way it was provided in each direction that—unless the size of the first step was set to a too large value which led to the increase of the partial derivative—the size of the second step was always smaller compared to the first step.

**Figure 3:**
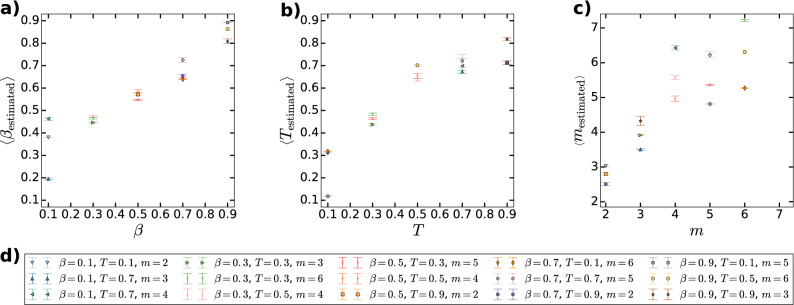
Estimated parameters in synthetic networks generated by the PSO model. We show the estimated values as a function of the true value used during the network generation process, averaged over 100 samples for the *m* parameter in panel (**a**), the $\beta$ parameter in panel (**b**), and the *T* parameter in panel (**c**). The colours indicate the different parameter combinations listed in panel (**d**). The bars indicate the 95% confidence intervals.

In order to validate our parameter inference method, in Fig. 3 we show the estimated $\beta$, *T* and *m* parameters as a function of the true parameter value used for generating synthetic networks of size $N=100$ according to the PSO model. Based on Fig. 3a, our framework yields estimated $\beta$ values that are quite close to the true values, except for the parameter combinations falling into the extremely low $\beta$ range. When moving to the results obtained for *T* shown in Fig. 3b, the deviation between $< T_{\mathrm{estimated}}>$ and the temperature *T* used during the network generation process is slightly larger; nevertheless, the estimated temperature is still not far from the true *T* value. A somewhat more scattered picture is shown for estimating *m* in Fig. 3c, where $< m_{\mathrm{estimated}}>$ is displaying a larger deviation compared to the previous cases. However, these estimations are still within the acceptable range. Based on these results, our parameter estimation framework shows a reasonably good performance when tested on synthetic networks with known model parameter values.

We have two important final remarks related to the parameters of the embedding, concerning the radial ordering of the nodes dictated by the node degrees. First, in the case of directed networks it is a natural idea to consider also the in- and the out-degree beside the total degree as potential candidates for determining the radial order. However, it is important to note that the ordering obtained in this way may become inconsistent with the likelihood maximisation that is derived inherently for undirected networks. Nevertheless, when considering model-independent quality scores, switching to the order dictated by either the in- or out-degree may also lead to an optimum that is impossible to reach when relying on the total degree. Therefore, here we take a practical approach by trying out all 3 possible radial orderings (determined by the total-, in- and out-degree) whenever dealing with directed networks and choose the ordering that yields the best quality scores.

Our second remark is related to the very likely ambiguity in the radial ordering for any network (both directed and undirected) caused by the occurrence of equal node degrees in the system. I.e., in real networks the degree distribution is usually skewed, meaning that a relatively large fraction of the nodes has a small degree compared to the average degree. This means that in the low degree regime we usually find a considerable number of nodes with the very same degree, hence the radial ordering dictated by Eqs. (11a–11b) allows actually a large number of different permutations within segments of the node ranking containing nodes with equal degree. According to our experiments detailed in the Supporting Information, there can be a non-negligible variance in the quality scores measuring the goodness of the embedding when permuting the radial order between nodes of the same degree for both HyperMap, the original ncMCE approach, and also the optimised ncMCE method proposed in this paper. Therefore, the actually chosen radial order (out of the many possibilities that are monotonic according to the degree) can be viewed as a further parameter of the embedding for the aforementioned methods. However, the optimal choice for this parameter can be set only via trial and error, i.e. by repeatedly trying out different random permutations between the nodes of the same degree, and keeping that radial order which produces the best quality score. Under some circumstances an estimate on the possible further improvement in the quality score as a function of the number of further tries can be made, as shown in the Supporting Information.

## Results

We have tested our method on both synthetic and real networks (our code is available from Ref.^42^). In order to quantify the quality of the embedding, we used the logarithmic loss defined in Eq. (12), and also the greedy routing score, which is a commonly used, model-free measure^34,36^. The idea of greedy routing on a network embedded in a geometric space corresponds to a simple routing protocol for getting from a source node *i* to a destination node *j* by walking on the network, where the next step from the current node is always carried out to the neighbour that is the closest to the destination *j* according to the distance measured in the given geometric space^43^. For networks embedded in a hyperbolic space, the distance we use during greedy routing is the hyperbolic distance between the nodes. In case a cycle is detected in the path, the routing protocol is unable to reach the destination and the path is stopped. Therefore, a natural simple measure for the success of the routing protocol is given by the fraction of successful paths actually reaching the destination without getting stuck on any other node^16,44^. In order to measure the success of the routing in a more refined way, we calculate the greedy routing score^36^ given by

14 $$\begin{aligned} GR = \frac{1}{N(N-1)}\sum \limits _{i=1}^N\sum \limits _{j=1,j\ne i}^N \frac{\ell _{ij}^{(SP)}}{\ell _{ij}^{(GR)}}, \end{aligned}$$

where $\ell _{ij}^{(SP)}$ denotes the shortest path length between *i* and *j*, and $\ell _{ij}^{(GR)}$ stands for the greedy routing path length between the same source-destination pair, which is considered to be infinity if the routing fails in reaching *j* from *i*.

**Figure 4:**
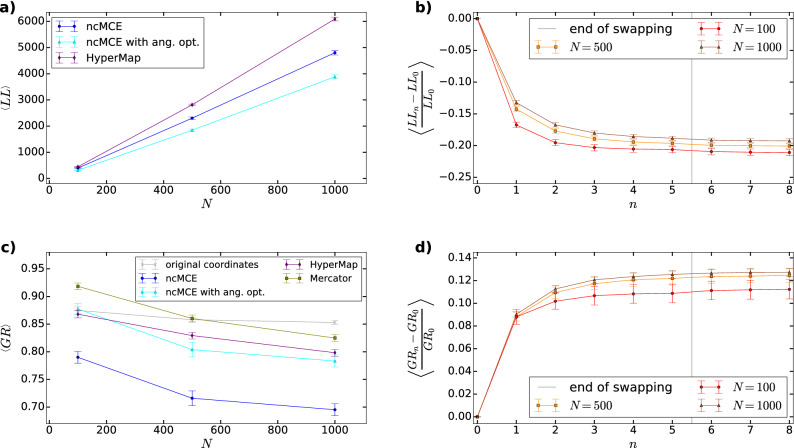
Embedding results for synthetic networks. (**a**) The average logarithmic loss $< LL>$ and the corresponding 95% confidence interval (indicated by bars) as a function of the number of nodes *N* for 100 networks generated by the PSO model using $\zeta =1$, $m=2,\beta =2/3$ and $T=0.3$. (**b**) The convergence of the logarithmic loss over the subsequent rounds of iterations during the proposed angular optimisation of the ncMCE method. (**c**) The average greedy routing score $< GR>$ and the corresponding 95% confidence interval (indicated by bars) as a function of the number of nodes *N* for the same synthetic data set as in panel (**a**). (**d**) The convergence of the greedy routing score as a function of the number of rounds *n* during our angular optimisation.

In Fig. 4 we show the results for synthetic networks generated by the PSO model with sizes $N=100,\,500$ and 1000. We tested four embedding methods on 100 networks at each network size. In Fig. 4a we plot the average logarithmic loss $< LL>$ as a function of *N* for HyperMap (purple), the original ncMCE (blue) and ncMCE with angular optimisation (cyan). (Since Mercator is based on the ${\mathbb {S}}^1/{\mathbb {H}}^2$ model, the logarithmic loss with respect to the E-PSO model cannot be considered as a fair quality function regarding this embedding method; therefore, Mercator is left out from Fig. 4a.) Not surprisingly, the curves show an increasing tendency with *N*; however, the angular optimisation clearly provides an about $20\%$ lower *LL* compared to ncMCE without optimisation, and about a $30\%$ lower value compared to HyperMap.

In Fig. 4b we show the relative change in *LL* as a function of the number of rounds *n* during our optimisation of the angular coordinates. According to the figure, the *LL* seems to settle to a more or less constant value after $6-8$ rounds. In Fig. 4c we display the average greedy routing score $< GR>$ as a function of the system size *N*. This figure indicates that the angular optimisation improves the result of ncMCE in terms of *GR* as well; however, the greedy routing score obtained with HyperMap is not surpassed, and the best greedy routing scores are obtained with Mercator. In Fig. 4d we plot the relative change in *GR* as a function of the number of rounds *n* in the angular optimisation of the result of ncMCE, where—similarly to Fig. 4b—a steady value is reached roughly above $n=6$. Additional figures related to embedding results on synthetic networks are provided in the Supporting Information.

In terms of real systems, we tested our method on the Pierre Auger collaboration network ($N=38$ nodes, available from Ref.^45^), a network between books about U.S. politics, where links correspond to frequent co-purchasing ($N=105$ nodes, available from Ref.^46^), the American College Football network ($N=115$ nodes, available from Ref.^47^), a Cambrian food web from the Burgess Shale ($N=142$ nodes, available from Ref.^41^), a protein interaction network from the PDZBase database ($N=161$ nodes, available from Ref.^48^) and a network of hyperlinks among a large set of U.S. political weblogs from before the 2004 election ($N=1222$ nodes, available from Ref.^49^). An important note about the Cambrian food web and the political blog network is that these networks are usually considered to be directed. According to that, we tried out all 3 options for defining the radial order among the nodes as described in the previous section for both HyperMap, the original ncMCE based coalescent embedding and our algorithm (whereas Mercator does not allow this option). Although the best quality scores were achieved with the total degree in the case of the political blog network, the comparison between the 3 options showed non-trivial results for the Cambrian food web, where the ordering according to the in-degree achieved the best greedy routing scores for both the original ncMCE approach and our algorithm, although its score was surpassed by the ordering according to the total degree in the case of HyperMap. More details on this aspect of the Cambrian food web are given in the Supporting Information.

**Figure 5:**
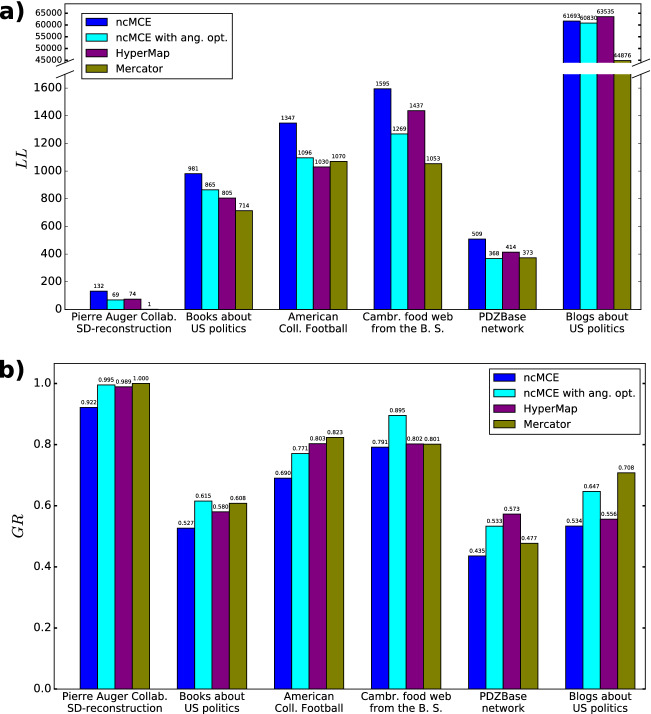
Embedding results for real networks. (**a**) The logarithmic loss for the original ncMCE (blue), ncMCE with angular optimisation (cyan), HyperMap (purple) and Mercator (olive) for the real networks that we studied. (**b**) The greedy routing score for the same methods.

In Fig. 5 we show a summary of the quality scores obtained for the real networks, displaying the best results we could achieve for each method depending on the choice of the embedding parameters. For each network of size $N<1000$ we performed embeddings trying out 2500 radial orders of the nodes with each $m-L-\beta -T$ parameter setting for HyperMap, the original ncMCE and our approach, and we also embedded each of these networks 2500 times with Mercator (where the repeated embedding of the same network also provides varying results). The political blog network was embedded 10 times with each method. The total number of rounds *n* needed in our optimisation framework varied between $n=8$ and $n=20$ for the studied real networks (details are given in the Supporting Information). In panel a) we compare the logarithmic loss *LL* for the different methods, where in the case of Mercator this was calculated according to the ${\mathbb {S}}^1$ model^13,38^ instead of the E-PSO model, and in panel b) we show the results for the greedy routing score *GR*. Further results at different parameter settings (together with a more detailed description of the studied networks) are given in the Supporting Information.

According to Fig. 5a, Mercator achieved the best *LL* score for 4 out of the 6 studied systems. However, this can be due to that some of these networks are more congruent with the ${\mathbb {S}}^1/{\mathbb {H}}^2$ model compared to the E-PSO model. When comparing only the methods building on the E-PSO model (that is HyperMap, the original ncMCE algorithm and our approach), our method obtained the lowest *LL* in 4 out of the 6 cases. If we narrow the scope further to the original ncMCE algorithm and our method, it seems that the angular optimisation is reducing the *LL* compared to the value obtained with the original ncMCE approach in all studied real networks. The maximum reduction was $47.6\%$ (for the Pierre Auger collaboration network) and the average reduction was $21.29\%$ for the studied systems. This reduction in the *LL* was necessary for bypassing the *LL* score of HyperMap in 3 networks, where the *LL* of the original ncMCE approach turned out to be higher compared to that of HyperMap.

When moving to the greedy routing score shown in Fig. 5b, we can see that the proposed optimisation of ncMCE improves the greedy routing score as well. The maximum improvement compared to the original ncMCE in terms of the *GR* was $22.5\%$ (for the protein interaction network from the PDZBase database), and the average improvement was $15.53\%$ for the studied systems. In addition, our approach achieved the highest *GR* for 2 out of the 6 studied networks, and the second best greedy routing score in 3 more cases. The overall best performance in terms of the *GR*-score in the studied real networks is achieved by Mercator, producing the highest value for 3 of the examined systems. At this point, it is important to note that although the *GR*-score allows a fair comparison between different methods in the sense that it is model-free, still, the intrinsic *GR*-score of real networks that could serve as the ground truth is unknown. According to that, a ranking between embedding methods based on the *GR*-score obtained in real networks should be treated with caution.

In the case of synthetic networks, the *GR*-score of the generated graph may be viewed as the ground truth, but the comparison becomes model-dependent, and the interpretation of cases where this ground-truth value is surpassed becomes somewhat ambiguous. By keeping in mind these limitations, in Fig. S3 in the Supporting Information we compare the *GR*-score of PSO networks with the *GR*-score of their embedding obtained with the different methods. The results indicate that the difference between the *GR*-score of a given embedding algorithm and the ground truth is highly dependent on the model parameters; thus, the induced ranking between the different approaches is also varying. Furthermore, all methods except the original ncMCE algorithm yielded a *GR*-score higher than the ground truth in several cases, adding an extra complication to the comparison. Although we cannot draw a conclusive ranking between the methods based on this analysis, the results clearly show that the *GR*-score for all of the studied methods is promisingly close to that of the ground truth graph for the majority of the parameter settings.

**Figure 6:**
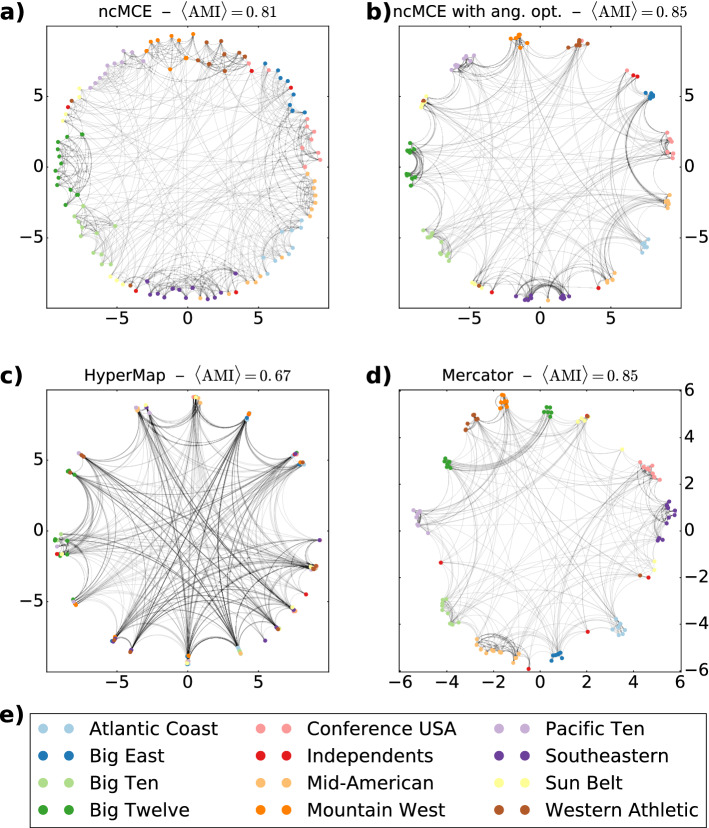
The layouts of the American College Football web on the native hyperbolic disk that reached the highest greedy routing scores. (**a**) The layout based on the coordinates resulted from the original ncMCE method. (**b**) The layout according to the coordinates obtained with our approach, optimising the results of ncMCE. (**c**) The hyperbolic layout obtained with HyperMap. (**d**) The embedding according to Mercator. The colour of the nodes indicates the team conference as listed in panel (**e**). The title of panels (**a**–**d**) include the average adjusted mutual information between the communities found by Louvain over 10 runs and the original team conferences.

In Fig. 6 we compare the layouts of the American College Football web in the 2D hyperbolic space obtained with the four different embedding methods. An interesting feature of this data set is that information about the conferences of the included teams is also available, which is marked by the different node colours in the figure. The angular coordinates of the nodes are equidistantly distributed in the output of the original ncMCE approach, as it can be seen in Fig. 6a. A visually quite pleasant feature of this layout is that according to the colouring, the teams belonging to the same conference tend to occupy a more or less well-defined, continuous range according to the angle. After applying the angular optimisation proposed in this paper, the angular coordinates are no longer equidistantly distributed and—as shown in Fig. 6b—the conferences contract into well-separated clusters, which helps the viewer even more in separating the different groups during a visual interpretation of the layout.

HyperMap seems to repeatedly assign the same (or very close) angular coordinates for multiple nodes at the same time, which results in very tight clusters in the layout (Fig. 6c); however, according to the colouring of the nodes, these clusters often contain nodes from different conferences. The layout obtained with Mercator (Fig. 6d) shows an organisation similar to that of our algorithm, where most of the team conferences appear as well-separated, but not extremely tight clusters.

In order to support the qualitative observations with quantitative measurements, we compared the modules forming according to the angular arrangement in the hyperbolic layouts in Fig. 6 to the “ground truth” clusters given by the team conferences in the data. A natural idea for locating these modules in an automated way is to apply a community finding method that can also take into account the hyperbolic distances. The angular separation of the communities seems to be a common feature of hyperbolic networks^50–53^, that on the one hand has inspired multiple generative models with an inherent community structure^32,54–56^, and on the other hand can be also exploited when the aim is to find the communities in a precise and efficient way^53^. Here we take a simple approach by applying such a version of the Louvain algorithm for community detection^57^ where the weighted modularity^58^

15 $$\begin{aligned} Q=\frac{1}{2M}\cdot \sum \limits _{i=1}^N\sum \limits _{j=1}^N \left[ w_{ij}-\frac{s_is_j}{2M}\right] \delta _{c_i,c_j}. \end{aligned}$$

optimised by the method was calculated according to link weights given by $w_{ij} = (1+x_{ij})^{-1}$ as suggested in Ref.^36^, the node strength $s_i$ was simply equal to $s_i = \sum _{\ell =1}^N w_{i\ell }$ and *M* denoted the total sum of the link weights. This way the 4 different layouts produced 4 different set of link weights over the same graph topology, and based on these we obtained 4 different partitioning of the network into communities with Louvain. These partitions were compared to the team conferences using the adjusted mutual information (*AMI*)^59^, corresponding to an information-theoretic similaritymeasure between sets of communities, where exact identity between the partitions results in $AMI=1$, and the similarity between random partitions is adjusted to $AMI=0$. The obtained *AMI* values are given above each layout in Fig. 6, and according to the results, the best *AMI* scores were achieved by our method and Mercator (both obtaining $< AMI>=0.85$ over 10 runs of the modularity optimisation), followed closely by the original ncMCE algorithm ($< AMI>=0.81$), with HyperMap somewhat separated from the rest (with $< AMI>=0.67$). These quantitative results are in full consistency with the qualitative observations detailed before.

**Figure 7:**
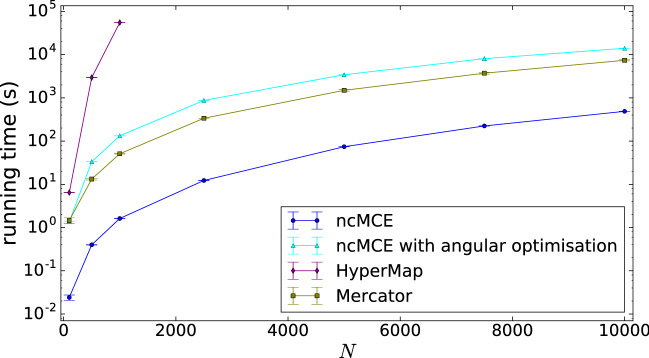
The running times of the studied embedding algorithms. The tests were run on synthetic networks obtained from the PSO model with $\zeta =1,\,m=2,\,\beta =2/3,\,T=0.3$ and *N* increasing from 100 to 10,000, where 10 samples were generated at each *N* value. The measured average running time is shown in seconds, the bars indicate the 95% confidence intervals.

In Fig. 7 we show the running time for the different embedding algorithms, measured for synthetic networks generated by the PSO model. The size of these networks varied between $N=100$ and $N=10,000$ nodes, while the further parameters of the network generating model were kept fixed.

The original ncMCE algorithm seems to be the fastest, with Mercator coming second followed quite closely by our approach, and HyperMap appears to be way slower compared to the rest at larger network sizes.

## Discussion

The coalescent hyperbolic embedding based on the ncMCE dimension reduction was shown to be a very efficient method with low running time and high-quality results^36^. In the present work we proposed a further optimisation of the angular coordinates obtained with this approach using a logarithmic loss function based on the E-PSO model. According to our experiments on both synthetic and real networks, this comes with the cost of a somewhat increased running time, but it also provides a lower logarithmic loss *LL* and a higher greedy routing score *GR*. The reduction of *LL* is not at all surprising (since we are actually optimising with regard to that); however, the improvement in *GR* in the meantime indicates that the embedding becomes better also according to a model-free quality score. In addition to the original ncMCE approach, we compared the results of our algorithm also with embeddings obtained with HyperMap^34^ and Mercator^38^, and apparently our algorithm is competing with these state-of-the-art methods in terms of the aforementioned two quality scores. In terms of the *GR*-score, Mercator seems to show the best overall performance; however, its advantage over our method is narrow, e.g. the best result is achieved in 3 real networks by Mercator, in 2 real networks by our approach and in 1 real network by HyperMap (where in addition, our method finished as the second, before Mercator). Although the *GR*-score is currently one of the most widely used quality scores in hyperbolic networks, it also has some limitations, e.g., the intrinsic *GR*-score of real networks that could serve as a ground truth is unknown. We discuss these issues in more details in the Supporting Information. In total, our studies of the quality scores achieved by the different embedding algorithms suggest that applying multiple different methods appears to be a good strategy when aiming for the highest quality embedding possible.

In the case of the American College Football web, the optimisation of the angular coordinates led to a result where clusters of nodes belonging to the same team conferences became more separated from the other groups compared to the layout in the original ncMCE approach. This shows that in some cases our algorithm not only improves the quality score of the embedding, but it can also provide a layout that is more intuitive and easy to interpret. Based on the above, the usage of our extension of the ncMCE coalescent embedding can be quite beneficial in any further study or application where high-quality hyperbolic embedding of networks is important.

A final remark we would like to make regarding the quality of the embedding (measured by either the logarithmic loss or the greedy routing score) is related to the radial order of the nodes dictated by the node degree in HyperMap, the original ncMCE coalescent embedding and also in our approach. As mentioned previously, real networks are very likely to contain (in some cases even large) groups of nodes with equal degree, and within such a group the radial order of the nodes can be chosen arbitrarily. According to our analysis (detailed in the Supporting Information), depending on the actual choice of the radial order the quality scores can show a non-negligible variance. Furthermore, in directed networks in principle we can choose from 3 degree types (corresponding to the in-, the out- and the total degree) when defining an ordering among the nodes. Although the radial order based on the in- or out-degrees may be inconsistent with the optimal radial order according to the (inherently undirected) hyperbolic model, our studies related to the Cambrian food web showed that the model-independent *GR*-score can still be higher for these alternative radial orderings. Therefore, these alternatives can help to find optimums that are not reachable from the standard radial ordering dictated by the undirected degree. Nevertheless, the systematic study of the embedding of directed networks is an interesting challenge for further work.

## Supplementary Information

Supplementary Information.
