# Supplementary material for: Optimisation of the coalescent hyperbolic embedding of complex networks
Source: Sci Rep. 2021 Apr 16;11:8350. doi: 10.1038/s41598-021-87333-5 (PMC8052422; doi:10.1038/s41598-021-87333-5)
Supplement: Supplementary file 1 — Supplementary Information. [file 41598_2021_87333_MOESM1_ESM.pdf]

Optimisation of the coalescent hyperbolic embedding of complex networks Supporting Information

Bianka Kovács[1] **and Gergely Palla**[1,2,3,*]

[1]Dept. of Biological Physics, Eötvös Loránd University, H-1117 Budapest, Pázmány P. stny. 1/A, Hungary
[2]MTA-ELTE Statistical and Biological Physics Research Group, H-1117 Budapest, Pázmány P. stny. 1/A, Hungary
[3]Health Services Management Training Centre, Semmelweis University, H-1125 Budapest, Kútvölgyi út 2, Hungary.
[*]pallag@hal.elte.hu

Embedding of synthetic networks generated by the PSO model

This section presents our results concerning synthetic networks. We compared the performance of the original ncMCE[1], ncMCE with our angular optimisation, HyperMap[2] and Mercator[3] for networks generated by the PSO model with different combinations of the input parameters. The effect of changing the number of nodes N is shown in the main article, while the dependence of the embedding quality measures on the expected average degree $2m$, the popularity fading parameter β and the temperature T used for the network generation is shown here below. An important remark is that both the logarithmic loss[2] LL and the greedy routing score[1] GR seem to converge after only a few rounds of angular optimisation for all types of synthetic networks that we studied, which is indispensable for keeping the running time of the angular optimisation low.

Besides, we demonstrate through the embedding of synthetic networks that for all the four investigated embedding methods the repeated embedding of the same network leads to hyperbolic arrangements of different quality. According to our experiments, the distribution of the embedding quality obtained by the repetition of a given method is a bell-shaped curve or consists of more bell-shaped peaks. Knowing this, we show how the improvement in both the logarithmic loss LL and the greedy routing score GR can be estimated after some trials as a function of the number of repetitions. The impact of the network generation parameters N, m, β and T on the variance of the embedding quality occurring during the repetition of the embedding is also investigated below.

The effect of the network generation parameters on the embedding quality

We tested four embedding methods (namely ncMCE, ncMCE with our angular optimisation, HyperMap and Mercator) on networks generated by the PSO model using different parameter combinations. The dependence of the logarithmic loss LL and the greedy routing score GR on the number of nodes N is depicted in the main article, and here in Figs. S1a, S1c, S1e and Figs. S2a, S2c, S2e we present the dependence of these quality measures on the half of expected average degree m, the popularity fading parameter β (or the expected exponent of the degree distribution $\gamma = 1 + 1/\beta$) and the temperature T (of which the expected average clustering coefficient is a decreasing function) used for network generation. A number of 100 networks were generated for each parameter setting. We then regarded the model parameters as unknown and set them for ncMCE, its optimisation and HyperMap according to the parameter optimisation method described in the main article. In the case of Mercator, we used its own parameter estimation process included in the algorithm[3]. Each network was embedded once with each embedding method and the obtained quality measures were averaged over the networks of the same generation parameters. For each network, we set two radial orders of the nodes: one for the parameter optimisation and – in order not to favour ncMCE over the other methods – another with which the performance of the ncMCE method was measured. This latter radial order was identical to the radial order used in our angular optimisation process and the radial order used in HyperMap. (Note that Mercator does not use a strict radial ordering among the network nodes.) According to Figs. S1b, S1d, S1f and Figs. S2b, S2d, S2f, after only 5 swapping and 3 non-swapping rounds of angular optimisation both the logarithmic loss LL and the greedy routing score GR reach a steady value for all type of PSO networks, which is in the case of LL $10-20\%$, while for GR $4-15\%$ better than the result of ncMCE without optimisation.

The GR-score in PSO networks

Probably the most popular score used to quantify the quality of the hyperbolic embedding of a complex network is given by the greedy routing score, as defined in the main text. A great advantage of this score is that it has no assumption about the

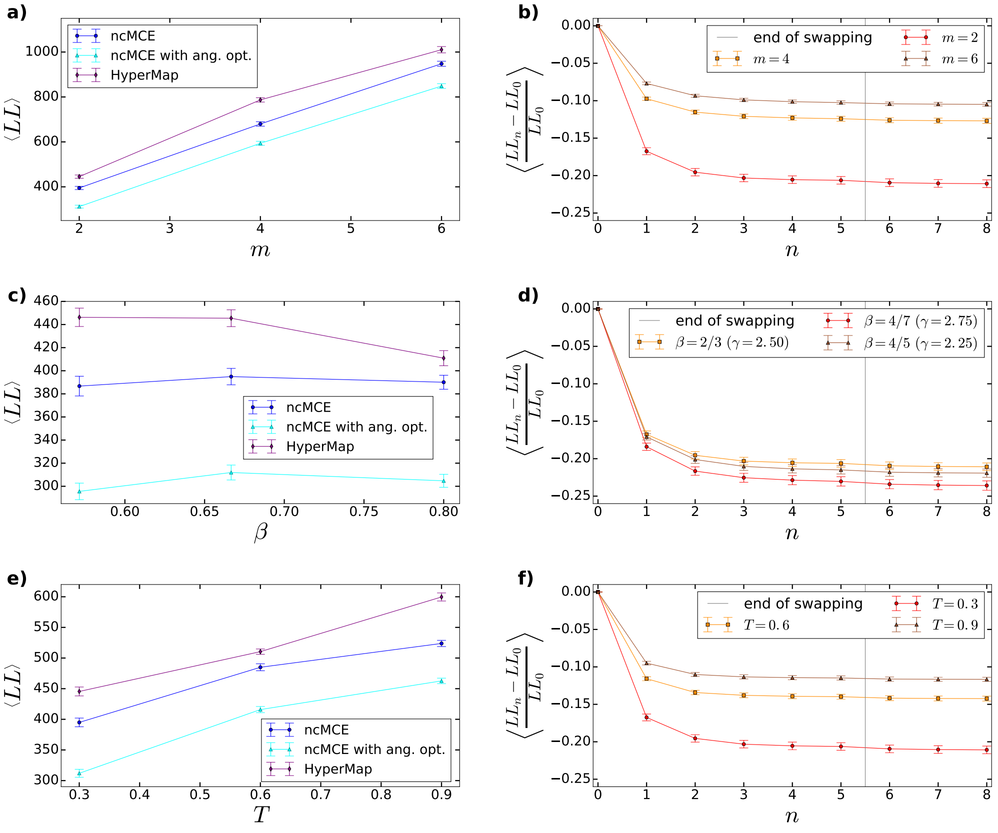

Figure S1. The impact of the network generation parameters on the logarithmic loss achieved by the original ncMCE, ncMCE with angular optimisation and HyperMap for networks generated by the PSO model. Logarithmic loss LL obtained with the studied embedding methods as a function of network generation parameters (left) and the relative improvement in the logarithmic loss as a function of the number of rounds n during the angular optimisation of the node arrangement resulted from the ncMCE method (right). Each data point corresponds to a value averaged over 100 synthetic networks, the bars indicate the 95% confidence intervals. a) LL as a function of the half of expected average degree m when $\zeta = 1$, $N = 100$, $\beta = 2/3$ and $T = 0.3$. b) Convergence of LL as a function of n under the same settings as in panel a). c) LL as a function of the popularity fading parameter β when $\zeta = 1$, $N = 100$, $m = 2$ and $T = 0.3$. d) The convergence of LL as a function of n under the same settings as in panel c). e) LL as a function of the temperature T when $\zeta = 1$, $N = 100$, $m = 2$ and $\beta = 2/3$. f) The convergence of LL as a function of n under the same settings as in panel e).

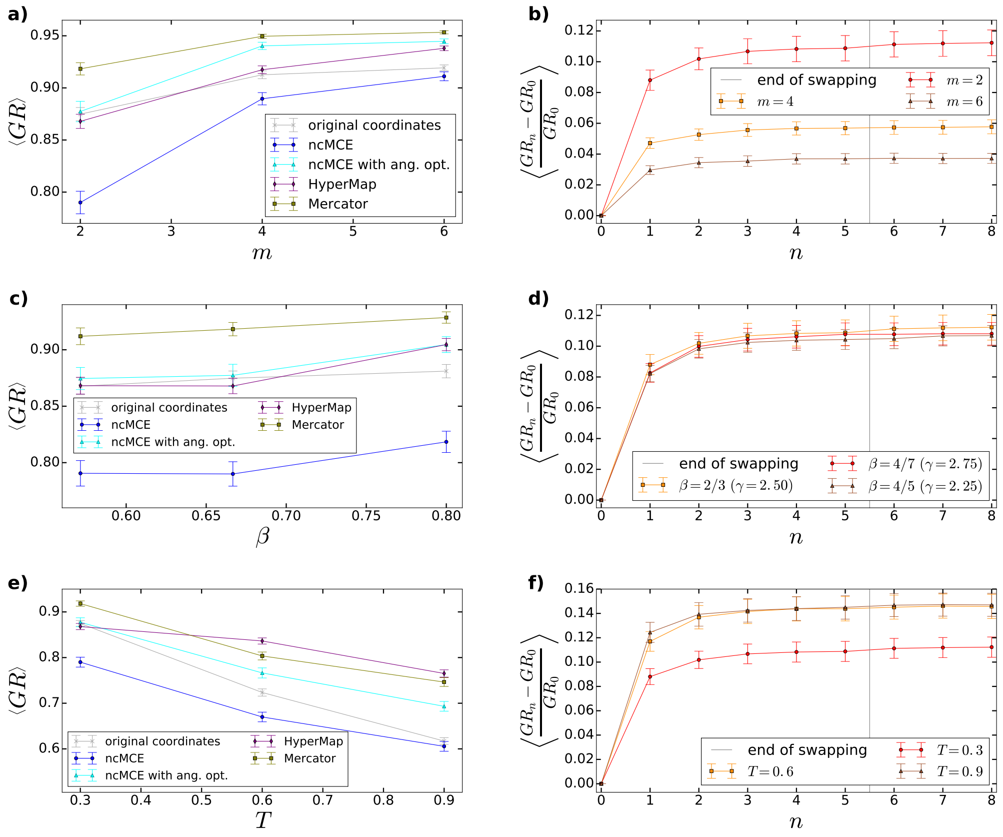

Figure S2. The impact of the network generation parameters on the greedy routing score achieved by the original ncMCE, ncMCE with angular optimisation, HyperMap and Mercator for networks generated by the PSO model. Greedy routing score *GR* obtained with the studied embedding methods as a function of network generation parameters (left) and the relative improvement in the greedy routing score as a function of the number of rounds n during the angular optimisation of the node arrangement resulted from the ncMCE method (right). Each data point corresponds to a value averaged over 100 synthetic networks, the bars indicate the 95% confidence intervals. a) *GR* as a function of the half of expected average degree m when $\zeta = 1$, $N = 100$, $\beta = 2/3$ and $T = 0.3$. b) Convergence of *GR* as a function of n under the same settings as in panel a). c) *GR* as a function of the popularity fading parameter β when $\zeta = 1$, $N = 100$, $m = 2$ and $T = 0.3$. d) The convergence of *GR* as a function of n under the same settings as in panel c). e) *GR* as a function of the temperature T when $\zeta = 1$, $N = 100$, $m = 2$ and $\beta = 2/3$. f) The convergence of *GR* as a function of n under the same settings as in panel e).

model that generated the studied network, enabling a fair comparison between embedding methods based on both the PSO model family and the $\mathbb{S}^1/\mathbb{H}^2$ model. However, in the case of the embedding of real networks the intrinsic *GR*-score of the given network is unknown and, because of this, one cannot judge whether the obtained score is close or far from this natural baseline. Nevertheless, when dealing with multiple embedding methods, in a simple practical "the larger the better" approach a ranking between the different algorithms can still be given according to the achieved *GR*-scores.

When considering e.g. PSO networks instead, the *GR*-score of the generated random graph can be treated as the ground truth value. The downside of such a comparison is that it becomes unfair against embedding algorithms based on the $\mathbb{S}^1/\mathbb{H}^2$ model (such as e.g. Mercator). Putting aside this issue for a moment, in Fig. S3 we collected the previously shown plots related to the *GR*-score in PSO networks in the column on the left (panels Fig. S3a, Fig. S3c, Fig. S3e and Fig. S3g), and prepared figures displaying the root-mean-square deviation (*RMSD*) between the *GR*-score achieved by the embedding algorithms and the ground truth, listed in the corresponding panels on the right (Fig. S3b, Fig. S3d, Fig. S3f and Fig. S3h). According to these, the *RMSD* is reasonably low for all methods for almost all of the examined parameter settings, ranging usually between 0.05 and 0.1. The actual ranking between the different algorithms according to the *RMSD* seems to be quite sensitive to the model parameters; thus, none of the examined methods can be announced to be a clear "winner" according to the lowest *RMSD* value.

Furthermore, it is important to note that according to the panels in the left column of Fig. S3, for all methods except the original ncMCE algorithm, we can observe cases where the *GR*-score of the embedded network is higher than that of the original ground truth graph. (It is quite plausible, that such a situation might occur for further parameter settings also in the case of the ncMCE approach). This makes the evaluation of the *RMSD* curves tricky since a higher *RMSD* value can indicate an actually better *GR*-score that would be more beneficial for e.g. practical applications compared to an embedding having a very low *RMSD* value, but accompanied by also a lower *GR*-score. There lies also a more abstract question behind this problem: In what aspect are we seeking an optimal hyperbolic embedding? When the goal is to lower the *RMSD* as much as possible, we are basically looking for embedding methods that yield results as close as possible to the model-generated networks we provided as inputs. Although this is an absolutely valid framework, it is important to keep in mind that for real networks usually we have no reason to prefer one hyperbolic model over another. Hence, a model-free approach such as seeking the maximal *GR*-score is also a valid alternative. By looking at Fig. S3, we can conclude that these alternative evaluation frameworks yield different results, that are moreover also model parameter dependent. This may seem disappointing from the point of view of the desire for a well-established ranking between the methods; nevertheless, the fact that all algorithms can yield both low *RMSD* values and high *GR*-scores is rather motivating.

Varying quality scores under the repetition of the embedding

This section sheds light on the fact that for all four embedding methods that we examined, the hyperbolic arrangements resulting from the repeated execution of the embedding may not be equivalent regarding their quality scores. We generated networks with the PSO model and embedded them repeatedly with the original ncMCE, ncMCE with angular optimisation, HyperMap and Mercator. In the former three cases, the quality differences between the repetitions are indicated by the changes both in the logarithmic loss *LL* and the greedy routing score *GR*, and the performance of Mercator – measured only by the greedy routing score *GR* – also varied under the repetitions.

The ncMCE method determines the radial coordinates of the network nodes in the logarithmic loss optimising way proposed originally as a part of the HyperMap method. As detailed in the main article, in order to minimise the logarithmic loss of the embedding with respect to the E-PSO model, HyperMap assigns logarithmically increasing radial coordinates to the nodes in the decreasing order of the node degrees. However, the radial order among the nodes of the same degree can not be determined analytically, and according to the commonly applied procedure the ties in the degree order can be broken arbitrarily. Due to this ambiguity in the radial ordering of the nodes, for the same network usually many different arrangements of the network nodes can be produced by HyperMap, the original ncMCE and ncMCE with angular optimisation. To study the effect of the allowed changes in the radial order of the nodes we embedded PSO networks with the aforementioned three methods multiple times, where the embeddings of a given network were carried out using in each repetition the same setting of the embedding parameters ζ, m, L, β and T (obtained with the logarithmic loss minimising method described in the main article), but a randomly chosen permutation of the radial order of nodes with equal degree. Mercator does not allow the specification of the radial order; thus, this algorithm was just simply re-run from scratch.

Figures S4a, S4c, S4e and Figures S5a, S4c, S4e exemplify that for synthetic networks generated by the PSO model the distribution of the embedding quality among the repetitions of the embedding is close to a Gaussian for the original ncMCE, ncMCE with angular optimisation and HyperMap. Note that the original ncMCE method is fundamentally different from its angularly optimised version and HyperMap in that respect, that in the former the angular coordinates are determined independently from the radial arrangement of the nodes, while in the latter two methods the angular arrangement depends on the actual radial node order. Thereby, it can be expected that the distribution of the embedding quality among the different radial orderings of the nodes is Gaussian for any embedding method which determines the radial coordinates the same way as

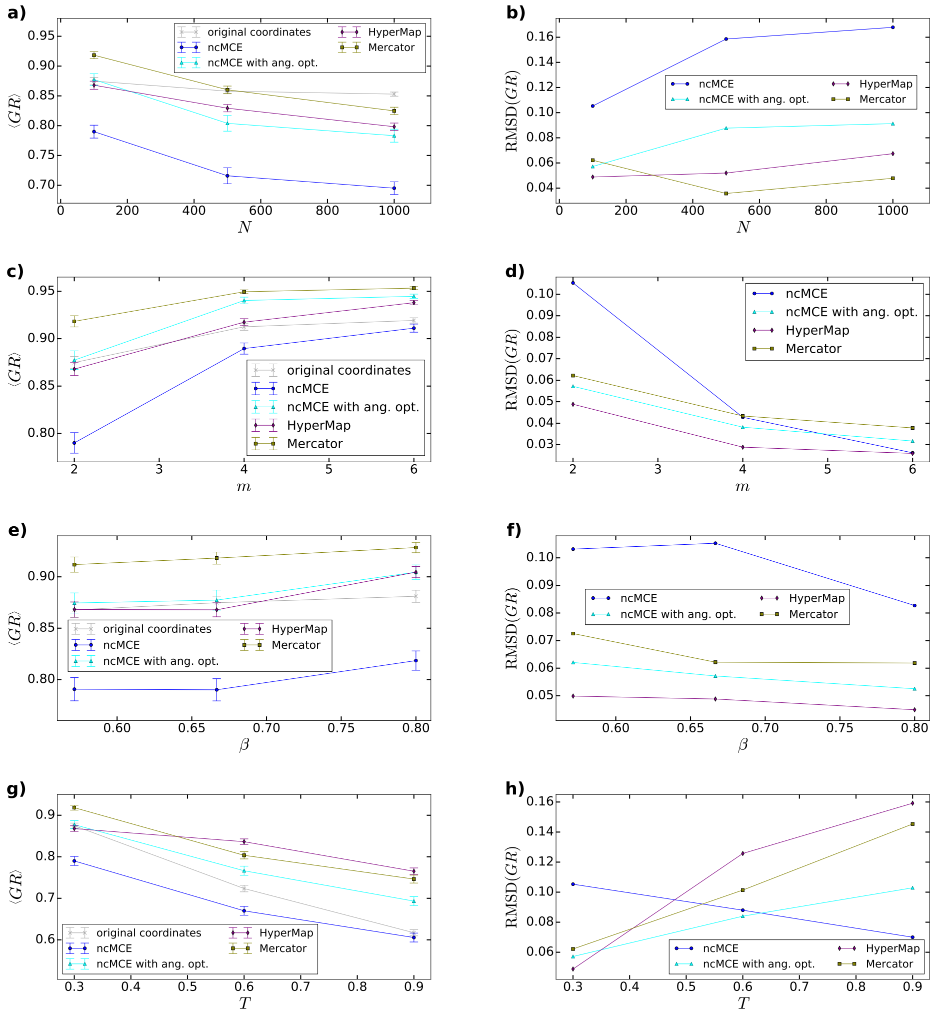

Figure S3. Comparing the *GR*-score of embedded networks and that of the original PSO graph. In panels a), c), e) and g) we reproduce the curves from Fig. 4c in the main paper and Fig. S2a, Fig. S2c, and Fig. S2e, displaying the average *GR*-score of the studied embedding methods together with the average *GR*-score of the generated PSO networks. Panels b), d), f) and h) show the corresponding root-mean-square deviation between the *GR*-score of the original PSO network and the *GR*-score of the embeddings obtained via the alternative methods.

HyperMap. Figure S5g demonstrates that the GR distribution obtained from the repeated execution of Mercator is not a simple normal distribution, but instead it consists of more bell-shaped peaks. However, if we are interested only in the best results achievable by repeating the embedding, we have to take into consideration only the peak at the end of the largest GR values, which in itself is similar to a normal distribution.

Assuming that the distribution of the quality score follows a normal distribution $\mathcal{N}(\mu,\sigma)$ (where μ is the mean and σ corresponds to the standard deviation), we can obtain an estimate for the best score that can be achieved under n_s number of repetitions of the embedding. The expected value for the extreme values of our interest among n_s number of samples can be formulated as

$$\mathbb{E}\left[\min_{1\le i\le n_s} LL_i\right] = \mu_{LL} - \sigma_{LL}\cdot \mathrm{g(n_s)}, \tag{S1a}$$

$$\mathbb{E}\left[\max_{1\le i\le n_s} GR_i\right] = \mu_{GR} + \sigma_{GR}\cdot \mathrm{g(n_s)}, \tag{S1b}$$

where n_s is assumed to be $n_s \geq 2$ and the function $g(n_s)$ is given by

$$g(n_s) = \sqrt{2\ln(n_s)} - \frac{\ln(\ln(n_s)) + \ln(4\pi) - 2\Gamma}{2\sqrt{2\ln(n_s)}} + \mathscr{O}\left(\frac{1}{\ln(n_s)}\right), \tag{S2}$$

where $\Gamma = 0.5772156649...$ is the Euler–Mascheroni constant[4]. We fitted a normal distribution to each of the measured quality score distributions (in the case of Mercator only to the relevant part of the distribution) and substituted the obtained μ and σ parameters into equations (S1a-S2). According to Figs. S4b, S4d, S4f and Figs. S5.b, S5d, S5f, S5h, the resulting functions are close to the curves describing the measured best quality score as a function of the number of embeddings carried out. This suggests that the improvement in the logarithmic loss or in the greedy routing score achievable by further repetitions of any of the four studied embedding methods can be predicted by simply fitting equations (S1a-S2) to the lowest achieved logarithmic loss or the highest achieved greedy routing score as a function of the number of trials so far.

We demonstrate through the original ncMCE method how the network properties can affect the variance of the embedding quality appearing when different radial orders of the nodes are tried out. We generated 500 networks with the PSO model using different parametrisations and embedded each network 250 times with ncMCE. The required embedding parameter β was determined for each network once by the $m-\beta-T$ optimising method described in the main article. During the repeated embedding of the same network only the radial order between the nodes having the same degree was allowed to change. Figures S6 and S7 show the results regarding the logarithmic loss and the greedy routing score of the embeddings, respectively. Besides, these PSO networks were also embedded 250 times with Mercator, the only one of the four studied embedding methods which does not set a strict radial order among the network nodes based on their degree. Figure S8 presents the improvement in the performance of Mercator due to its repeated execution in the case of several different parametrisations of the network generation.

Embedding of real networks

This section details our results related to real networks. We begin with the description of the adjustment of the embedding parameters, which is followed by the results obtained for the quality scores at different parameter settings. In addition, we show how the repetition of the embedding procedure can improve the achieved quality scores. Finally, some layouts of the networks in the native representation of the hyperbolic plane are also presented.

Parameter settings for the studied networks

We studied the following real networks:

- the twelfth layer of the multiplex Pierre Auger collaboration network[5], which describes the collaborations related to the SD-reconstruction;
- a network[6] between books about U.S. politics published close to the 2004 U.S. presidential election, where links correspond to frequent co-purchasing by the same buyers and nodes have been given values "l", "n", or "c" to indicate whether they are "liberal", "neutral", or "conservative";
- the American College Football network[7] describing the games between Division IA colleges during regular season Fall 2000, where the nodes have values that indicate to which conferences they belong;

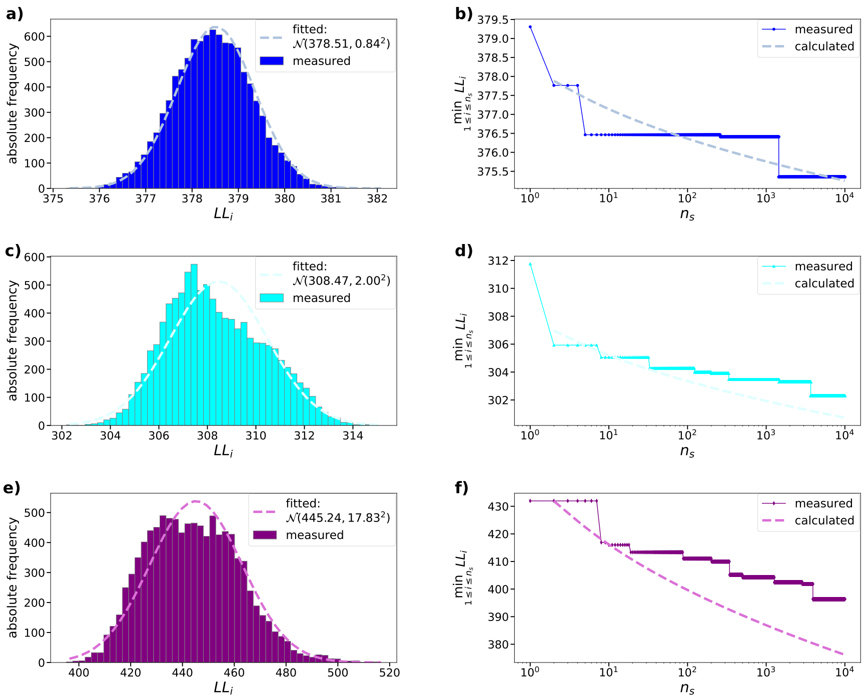

Figure S4. The distribution of the logarithmic loss LL among the repeated embeddings of a network generated by the PSO model parametrised by $\zeta = 1$, $N = 100$, $m = 2$, $\beta = 2/3$ and $T = 0.3$. In each row of the figure the results regarding one of the studied embedding algorithms are presented: panels a) and b) refer to ncMCE, panels c) and d) refer to ncMCE with our angular optimisation and panels e) and f) refer to HyperMap. The left panels show the observed quality distributions and the normal distributions fitted to these data, while the right panels depict the achieved best logarithmic loss as a function of the number of repetitions of the embedding. The dashed curves on the right are obtained from equation (S1a) by substituting in the mean and the standard deviation of the corresponding fitted normal distribution on the left.

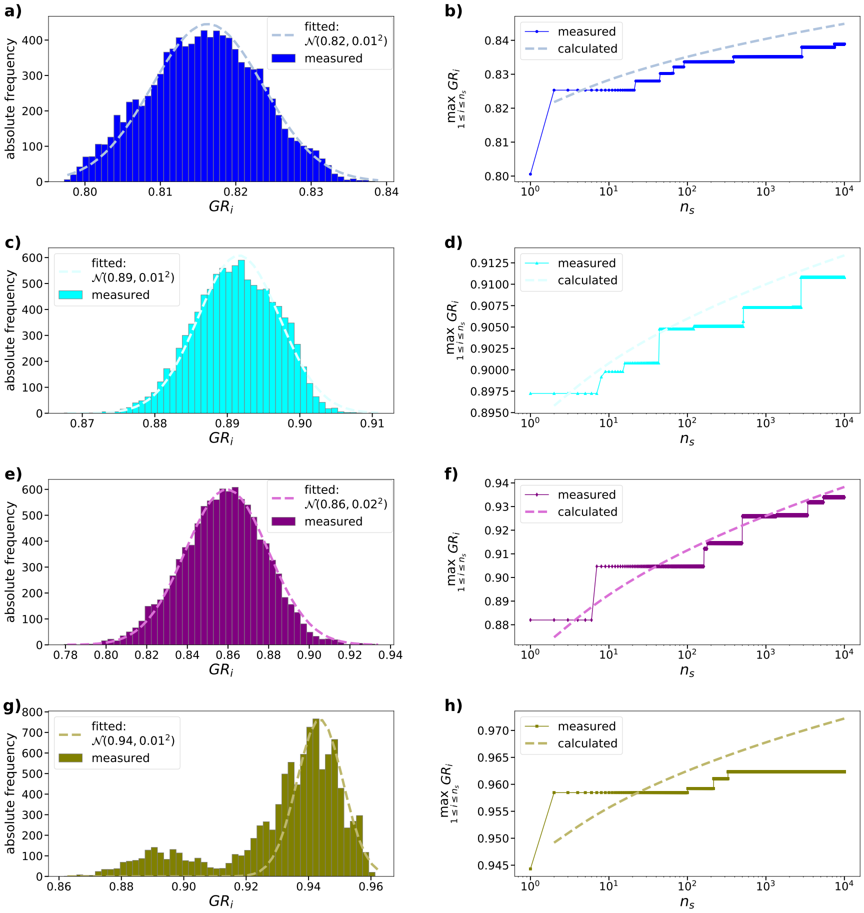

Figure S5. The distribution of the greedy routing score GR among the repeated embeddings of a network generated by the PSO model parametrised by $\zeta = 1$, $N = 100$, $m = 2$, $\beta = 2/3$ and $T = 0.3$. In each row of the figure the results regarding one of the studied embedding algorithms are presented: panels a) and b) refer to ncMCE, panels c) and d) refer to ncMCE with our angular optimisation, panels e) and f) refer to HyperMap and panels g) and h) refer to Mercator. The left panels show the observed quality distributions and the normal distributions fitted to these data, while the right panels depict the achieved best greedy routing score as a function of the number of repetitions of the embedding. The dashed curves on the right are obtained from equation (S1b) by substituting in the mean and the standard deviation of the corresponding fitted normal distribution on the left.

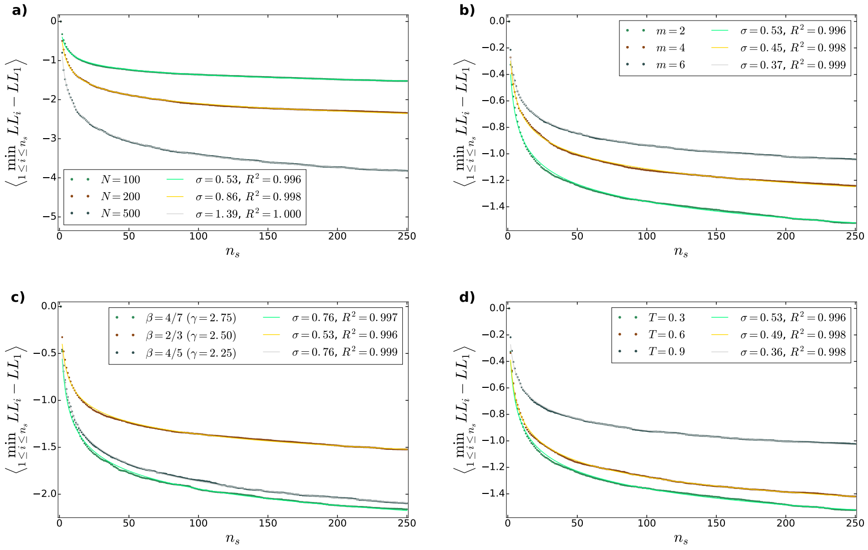

Figure S6. The impact of the network generation parameters on the variance of the logarithmic loss LL observed during the repeated execution of ncMCE for networks generated by the PSO model. The depicted data points correspond to the improvement in the embedding quality averaged over 500 PSO networks generated using a given parameter setting. The solid lines are obtained by fitting equation (S1a) to the measured curves. The fitted coefficient is the standard deviation σ characterising the distribution of the improvement in the logarithmic loss compared to the first trial of the embedding. Note that since not the absolute values, but the improvements are plotted, the mean of the quality distribution is always 0. The coefficient of determination R^2 is also given for each fit in the legends. a) The improvement in LL as a function of the number of repetitions of the embedding at different N parameters with fixed $\zeta = 1$, $m = 2$, $\beta = 2/3$ and $T = 0.3$. b) The improvement in LL as a function of the number of repetitions of the embedding at different m parameters with fixed $\zeta = 1$, $N = 100$, $\beta = 2/3$ and $T = 0.3$. c) The improvement in LL as a function of the number of repetitions of the embedding at different β parameters with fixed $\zeta = 1$, $N = 100$, $m = 2$ and $T = 0.3$. d) The improvement in LL as a function of the number of repetitions of the embedding at different T parameters with fixed $\zeta = 1$, $N = 100$, $m = 2$ and $\beta = 2/3$.

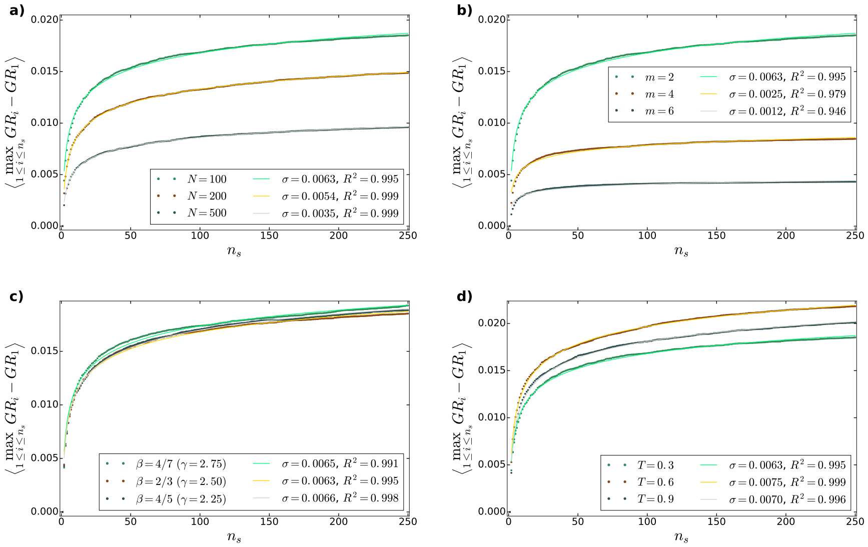

Figure S7. The impact of the network generation parameters on the variance of the greedy routing score *GR* observed during the repeated execution of ncMCE for networks generated by the PSO model. The depicted data points correspond to the improvement in the embedding quality averaged over 500 PSO networks generated using a given parameter setting. The solid lines are obtained by fitting equation (S1b) to the measured curves. The fitted coefficient is the standard deviation σ characterising the distribution of the improvement in the greedy routing score compared to the first trial of the embedding. Note that since not the absolute values, but the improvements are plotted, the mean of the quality distribution is always 0. The coefficient of determination R^2 is also given for each fit in the legends. a) The improvement in *GR* as a function of the number of repetitions of the embedding at different N parameters with fixed $\zeta = 1$, $m = 2$, $\beta = 2/3$ and $T = 0.3$. b) The improvement in *GR* as a function of the number of repetitions of the embedding at different m parameters and fixed $\zeta = 1$, $N = 100$, $\beta = 2/3$ and $T = 0.3$. c) The improvement in *GR* as a function of the number of repetitions of the embedding at different β parameters and fixed $\zeta = 1$, $N = 100$, $m = 2$ and $T = 0.3$. d) The improvement in *GR* as a function of the number of repetitions of the embedding at different T parameters and fixed $\zeta = 1$, $N = 100$, $m = 2$ and $\beta = 2/3$.

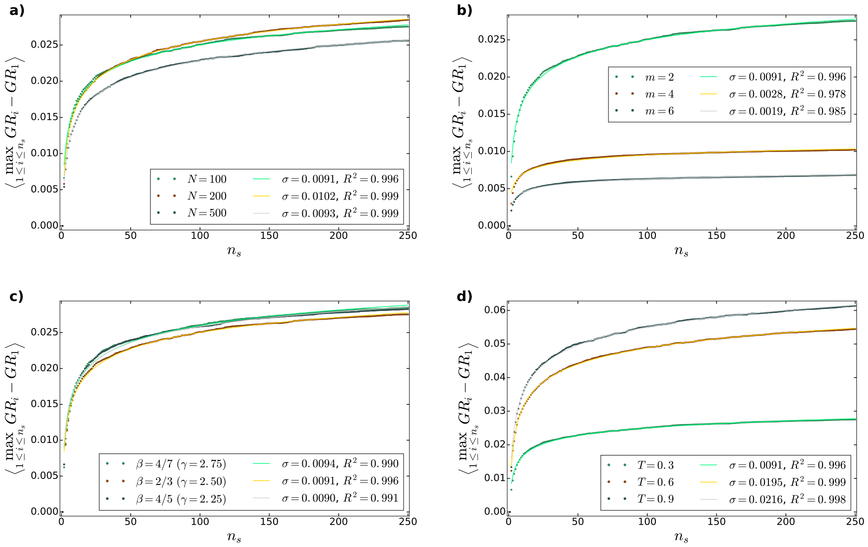

Figure S8. The impact of the network generation parameters on the variance of the greedy routing score *GR* observed during the repeated execution of Mercator for networks generated by the PSO model. The depicted data points correspond to the improvement in the embedding quality averaged over 500 PSO networks generated using a given parameter setting. The solid lines are obtained by fitting equation (S1b) to the measured curves. The fitted coefficient is the standard deviation σ characterising the distribution of the improvement in the greedy routing score compared to the first trial of the embedding. Note that since not the absolute values, but the improvements are plotted, the mean of the quality distribution is always 0. The coefficient of determination R^2 is also given for each fit in the legends. a) The improvement in GR as a function of the number of repetitions of the embedding at different N parameters with fixed $\zeta = 1$, $m = 2$, $\beta = 2/3$ and $T = 0.3$. b) The improvement in GR as a function of the number of repetitions of the embedding at different m parameters and fixed $\zeta = 1$, $N = 100$, $\beta = 2/3$ and $T = 0.3$. c) The improvement in GR as a function of the number of repetitions of the embedding at different β parameters and fixed $\zeta = 1$, $N = 100$, $m = 2$ and $T = 0.3$. d) The improvement in GR as a function of the number of repetitions of the embedding at different T parameters and fixed $\zeta = 1$, $N = 100$, $m = 2$ and $\beta = 2/3$.

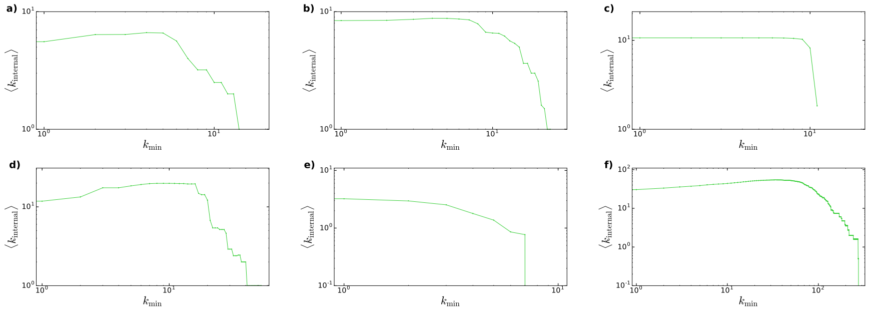

Figure S9. Average internal degree of the subgraph spanning between nodes having a degree $k > k_{\min}$ as a function of the degree threshold $k_{\min}$ for the studied real networks. a) The twelfth layer of the Pierre Auger collaboration network. b) Network between books about U.S. politics. c) American College Football network. d) Cambrian food web from the Burgess Shale. e) Protein interaction network from the PDZBase database. f) Network between blogs about U.S. politics.

- a Cambrian food web from the Burgess Shale[8] with nodes categorised according to their trophic roles, which is in fact a directed network where links are pointing from consumers to resources, but in order to enable the hyperbolic embedding, the directedness of the connections can be disregarded;
- a protein interaction network[9] from the PDZBase database;
- a network[10] of hyperlinks between blogs on U.S. politics from before the 2004 election, where the nodes have been given values 0 or 1 to indicate whether they are left/liberal or right/conservative.

Their characteristics related to the parameters of the logarithmic loss, namely the number of nodes N, the average degree $\langle k \rangle$, the smallest occurring degree $\min_{1\leq i\leq N} k_i$ and the average clustering coefficient $\langle c \rangle$ are listed in Table S1. Because of the small network sizes, fitting the degree decay exponent γ is ambiguous in most cases; therefore, γ values are not provided. As it was pointed out in the section of the main article devoted to the E-PSO model, the parameter L is related to the shape of the curve describing the connection between a degree threshold and the average internal degree of the subgraph determined by this threshold. Figure S9 displays this curve for the studied real networks.

At least two different settings of the embedding parameters were tested for each real network in the case of ncMCE, its angular optimisation and HyperMap. First, the same procedure was carried out as in the case of synthetic networks: the parameters m, β and T were determined simultaneously by minimising the logarithmic loss LL of an embedding resulted from the ncMCE method and L was calculated as $\langle k \rangle /2 - m$. In the second and third cases of the parameter setting, the parameters β and T were optimised in a similar way as in the first case, but here the parameter m was previously fixed to $\langle k \rangle /2$ and $\min_{1\leq i\leq N} k_i$, respectively; thus, instead of the 3-dimensional gradient $(\frac{\partial LL}{\partial m}, \frac{\partial LL}{\partial \beta}, \frac{\partial LL}{\partial T})$ the 2-dimensional vector $(\frac{\partial LL}{\partial \beta}, \frac{\partial LL}{\partial T})$ was used. Setting m to $\langle k \rangle /2$ corresponds to the assumption that the network was generated by the original PSO model, while the choice $m = \min_{1\leq i\leq N} k_i$ was proposed for HyperMap[2]. We did not embed the American College Football network with $m = \min_{1\leq i\leq N} k_i$, because this would have resulted in a negative $L = \langle k \rangle /2 - m$ value which was originally not allowed in HyperMap. For the Cambrian food web and the network of political blogs (in which the links are actually directed) the type of the node degree determining the radial order of the nodes was considered to be an additional parameter of the embedding: in the fourth and the fifth parameter setting procedures the simultaneous optimisation of m, β and T was carried out using such a radial node order that resulted in outwards decreasing in-degree and out-degree, respectively. All the parameter settings tested for ncMCE, its angular optimisation and HyperMap are given in Table S1. In the case of Mercator, we used its own parameter estimation process included in the algorithm[3].

Embedding quality at different parameter settings

In the following, we show detailed results regarding the behaviour of the quality scores as a function of the embedding parameters. As it can be seen in Fig. S10, both the logarithmic loss LL and the greedy routing score GR reach a more or less steady value for all the studied real networks and parameter settings within a reasonable number of rounds of angular optimisation of the node arrangement obtained with ncMCE, just like in the case of synthetic networks.

In the previous section we have shown that for synthetic networks the achievable improvement in the quality scores under the repetition of the embedding can be predicted for all the four studied embedding methods by fitting the formulae in equations (S1a-S2) to the lowest achieved LL or the highest achieved GR as a function of the number of trials so far. In Fig. S11 we

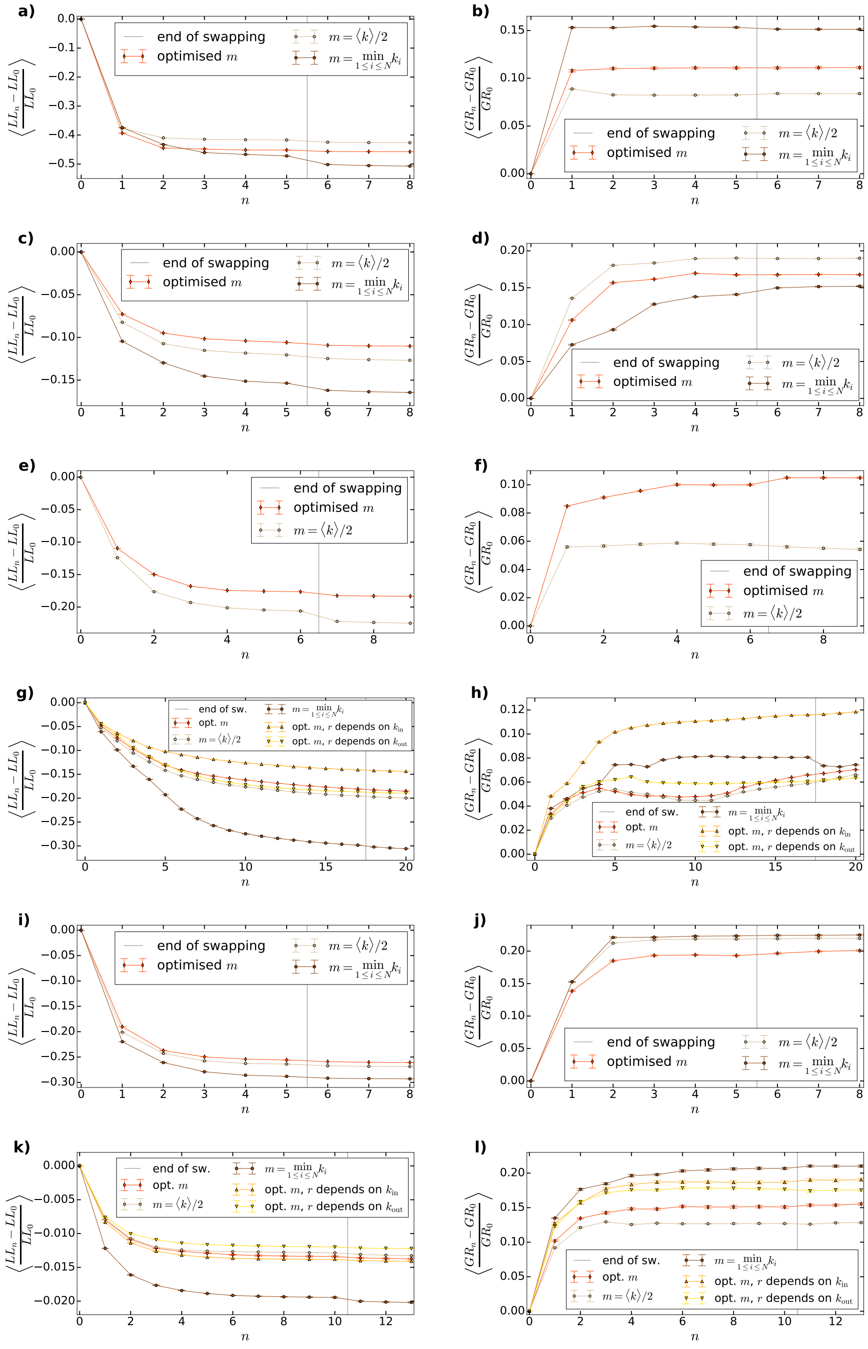

Figure S10. **The convergence of the logarithmic loss LL (left) and the greedy routing score GR (right) over the subsequent rounds of iterations during the angular optimisation of the embeddings resulted from ncMCE for the studied real networks.** The curves of different colours show the relative improvement in the embedding quality for different settings of the embedding parameters: either m was optimised simultaneously with β and T via gradient descent, or only β and T were optimised based on the logarithmic loss and m was set to the half of average degree or the smallest occurring degree. In the case of the Cambrian food web (panels g) and h)) and the network of political blogs (panels k) and l)) two further parameter adjusting procedures were also carried out, where the parameter m was optimised simultaneously with β and T for such node arrangements where the radial coordinates were assigned in the order of the in- or the out-degrees instead of the total degrees of the nodes. With the exception of the network of political blogs where only 10 embedding trials were carried out for each parameter setting, each data point corresponds to an average over 2500 trials of embedding with the given m, β and T parameters, and the bars indicate the 95% confidence intervals. The total number of optimisation rounds was set for each network individually to a value at which the logarithmic loss seemed to settle to a more or less constant value with all the examined parameter settings. Panels a) and b): The twelfth layer of the Pierre Auger collaboration network. Panels c) and d): Network between books about U.S. politics. Panels e) and f): American College Football network. Panels g) and h): Cambrian food web from the Burgess Shale. Panels i) and j): Protein interaction network from the PDZBase database. Panels k) and l): Network between blogs about U.S. politics.

Table S1. Properties of the studied real networks and the embedding parameters used for ncMCE, its angular optimisation and HyperMap. The following observed network characteristics are listed: the number of nodes N, the average degree $\langle k\rangle$, the minimal degree $\min_{1\le i\le N} k_i$ and average clustering coefficient $\langle c\rangle$. For each network at least two different procedures were carried out for setting the embedding parameters m, L, β and T. The main idea behind our parameter estimation approach was to minimise the logarithmic loss LL of an embedding obtained with the ncMCE method. Note that ζ was always set to 1, which corresponds to the standard practice in the literature, and L was actually not considered to be a free parameter, instead it was always calculated as $L = \langle k\rangle/2 - m$.

	P. A. Collab. SD-reconstruction	Books about US politics	American College Football	Cambrian food web from the B. S.	Prot. int. network from PDZBase	Blogs about U.S. politics
**Measured network characteristics**						
N	38	105	115	142	161	1222
$\langle k\rangle$	5.368	8.4	10.661	10.761	2.596	27.36
$\min_{1\le i\le N} k_i$	1	2	7	1	1	1
$\langle c\rangle$	0.806	0.488	0.403	0.205	0.007	0.320
**Optimal embedding parameters according to a gradient descent in the $m-\beta-T$ space with $\langle k\rangle/2 < m$ allowed**						
m	3.067	5.437	10.965	6.233	1.524	12.582
L	−0.383	−1.237	−5.635	−0.853	−0.226	1.096
β	0.561	0.526	0.1	0.979	0.591	0.679
T	0.347	0.624	0.577	0.804	0.536	0.696
**Optimal embedding parameters according to a gradient descent in the $\beta-T$ space using $m=\langle k\rangle/2$ and $L=0$**						
β	0.618	0.579	0.304	0.888	0.634	0.588
T	0.379	0.590	0.588	0.747	0.519	0.594
**Optimal embedding parameters according to a gradient descent in the $\beta-T$ space using $m=\min_{1\le i\le N} k_i$ and $0<L=\langle k\rangle/2-m$**						
β	0.939	0.762	–	0.99	0.688	0.657
T	0.504	0.621	–	0.664	0.543	0.631
**Optimal embedding parameters according to a gradient descent in the $m-\beta-T$ space with $\langle k\rangle/2 < m$ allowed when the radial order is determined by sorting the nodes according to their in-degree k_{in}**						
m	–	–	–	9.976	–	12.203
L	–	–	–	−4.596	–	1.475
β	–	–	–	0.681	–	0.797
T	–	–	–	0.853	–	0.825
**Optimal embedding parameters according to a gradient descent in the $m-\beta-T$ space with $\langle k\rangle/2 < m$ allowed when the radial order is determined by sorting the nodes according to their out-degree k_{out}**						
m	–	–	–	8.642	–	13.013
L	–	–	–	−3.262	–	0.665
β	–	–	–	0.740	–	0.713
T	–	–	–	0.837	–	0.835

demonstrate that although in the case of real networks it is often not true anymore that the quality distribution among the repetitions of the embedding is a simple normal distribution, the corresponding formulae in equations (S1a-S2) still can be used to fit the observed curves of the best quality scores achieved so far. The fitted coefficients μ and σ correspond to the mean and the standard deviation of the peak found on that side of the quality distribution which yields the best results. Thus, it seems that fitting equations (S1a-S2) automatically selects the part of the quality distribution that is relevant for estimating the quality improvement achievable by the repetition of the embedding.

We embedded the five real networks of size $N < 1000$ with each of the examined embedding methods 2500 times. In the case of ncMCE, ncMCE with angular optimisation and HyperMap we set the embedding parameters only once for each network with each of the above-described parameter estimating methods and during the repetition of the embedding only the radial order of the nodes having the same degree was randomly permuted again and again. Since Mercator has its own parameter

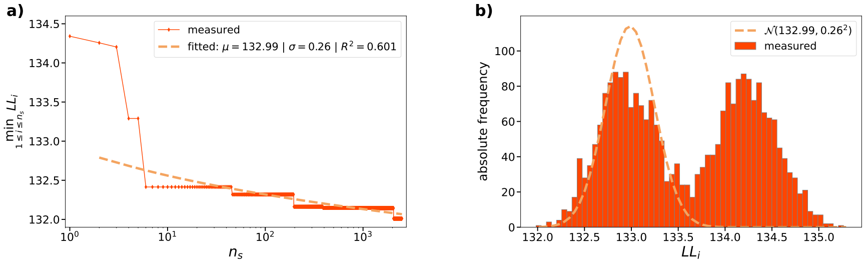

Figure S11. Example for fitting to the achieved best logarithmic loss as a function of the number of embedding trials for a real network where the distribution of the obtained quality scores among the repetitions of the embedding seems to be bimodal. The here considered LL values resulted from the repeated embedding of the Pierre Auger collaboration network using the original ncMCE approach with the parameters m, β and T obtained from our parameter optimisation procedure described in the main article. a) The achieved best LL values as a function of the number of samples n_s, together with the fitted curve according to equations (S1a) and (S2). b) The density function of the obtained LL scores (shown by the histogram) together with a normal distribution (dashed line) having a mean and standard deviation corresponding to the fit shown in panel a), re-normalised according to the weight of the empirical LL samples below $LL = 133$.

estimating process and does not allow the setting of the radial order of the nodes, Mercator was simply re-run 2500 times. The achieved best quality scores are plotted as a function of the number of repetitions n_s in Figs. S12-S16. We fitted to each curve according to equations (S1a-S2), the fitted coefficients are listed in Tables S2-S6. An interesting point to note related to these results is that the greedy routing score achieved for the Cambrian food web was way higher when the radial ordering of the nodes was dictated by the in-degree instead of the out-degree or the total degree for both the original ncMCE approach and its angularly optimised version.

Table S2. The results of fitting the formulae in equations (S1a-S2) to the curves plotted in Fig. S12 showing the achieved best quality scores as a function of the number of embeddings carried out in the case of the twelfth layer of the Pierre Auger collaboration network. The fitted coefficients are the mean (μ_{LL} and μ_{GR}) and the standard deviation (σ_{LL} and σ_{GR}) characterising the peak on that side of the quality score's observed distribution which corresponds to the best results. The quality of the fits is characterised by the coefficient of determination (R^2_{LL} and R^2_{GR}), which is a sufficiently high value in most of the cases. For the three embedding methods that were tried out with various parametrisations, the results regarding the eventually (after 2500 repetitions) best parameter setting are written in bold.

Embedding method	μ_{LL}	σ_{LL}	R^2_{LL}	μ_{GR}	σ_{GR}	R^2_{GR}
ncMCE, optimised m	**132.99**	**0.261**	**0.60**	0.906	0.0016	0.87
ncMCE, $m = \langle k \rangle /2$ and $L = 0$	141.70	0.157	0.79	**0.910**	**0.0033**	**0.67**
ncMCE, $m = \min_{1\le i\le N} k_i$ and $0 < L = \langle k \rangle /2 - m$	204.91	0.202	0.77	0.851	0.0014	0.49
ncMCE with ang. opt., optimised m	**70.29**	**0.296**	**0.34**	**0.994**	**0.0003**	**0.05**
ncMCE with ang. opt., $m = \langle k \rangle /2$ and $L = 0$	79.75	0.738	0.77	0.993	0.0006	0.06
ncMCE with ang. opt., $m = \min_{1\le i\le N} k_i$ and $0 < L = \langle k \rangle /2 - m$	100.56	3.020	0.89	0.986	0.0017	0.54
HyperMap, optimised m	80.69	0.795	0.65	0.934	0.0124	0.73
HyperMap, $m = \langle k \rangle /2$ and $L = 0$	**79.14**	**1.369**	**0.86**	**0.965**	**0.0071**	**0.65**
HyperMap, $m = \min_{1\le i\le N} k_i$ and $0 < L = \langle k \rangle /2 - m$	81.54	1.381	0.43	0.977	0.0026	0.40
Mercator	–	–	–	0.999	0.0003	0.11

Hyperbolic layouts for real networks

In addition to the hyperbolic layouts of the American College Football web presented in the main article, here we show layouts on the native representation of the hyperbolic plane also for the network between books about U.S. politics and the Cambrian food web from the Burgess Shale. Similarly to the American College Football network, the nodes in these two further networks form communities. In the case of the network between books about U.S. politics, shown in Fig. S17, the communities are based on political orientation, whereas for the Cambrian food web, depicted in Fig. S18, the nodes can be sorted into different trophic roles. For both networks, we have chosen the layout corresponding to the achieved best greedy routing score for each

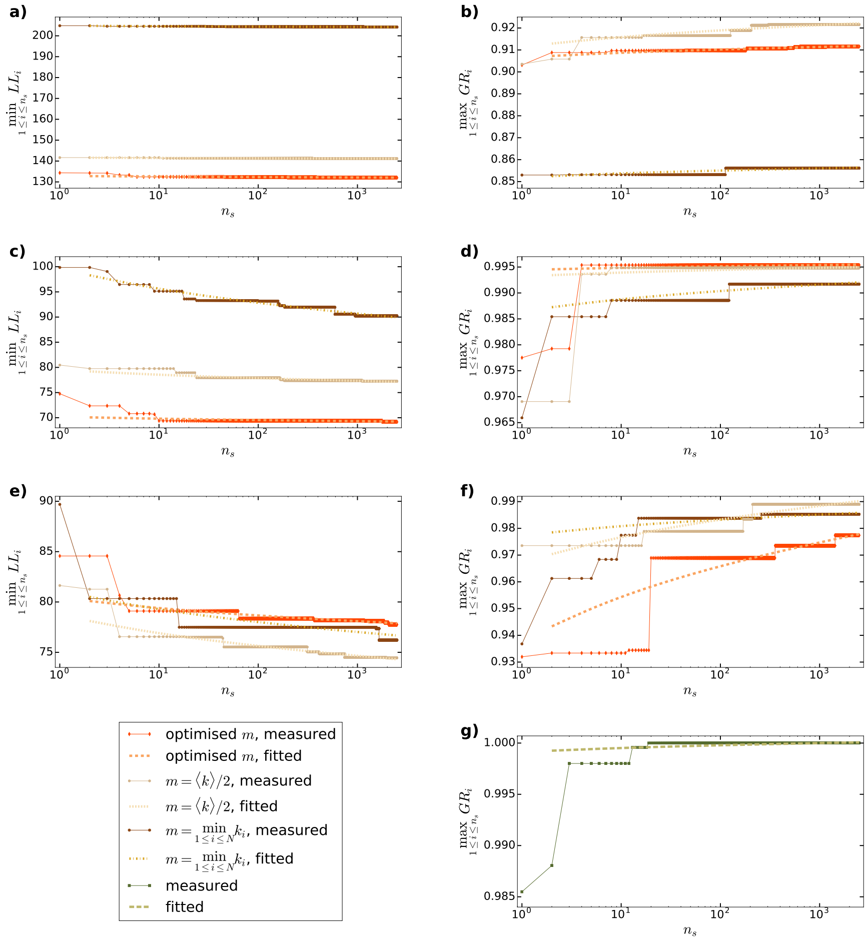

Figure S12. The achieved best logarithmic loss (left) and greedy routing score (right) as a function of the number of repetitions of the embedding in the case of the twelfth layer of the Pierre Auger collaboration network. Each row of the figure presents the results regarding one of the studied embedding algorithms: panels a) and b) refer to ncMCE, panels c) and d) refer to ncMCE with angular optimisation, panels e) and f) refer to HyperMap and panel g) refers to Mercator. Excepting Mercator, all the embedding methods were tested using 3 different parameter settings: either m was optimised together with β and T, or only β and T were optimised with m fixed to the half of average degree or the smallest occurring degree. The dashed curves were obtained by fitting the corresponding formulae of equations (S1a-S2) to the solid curves. The fitted coefficients and the quality of the fits are listed in Table S2.

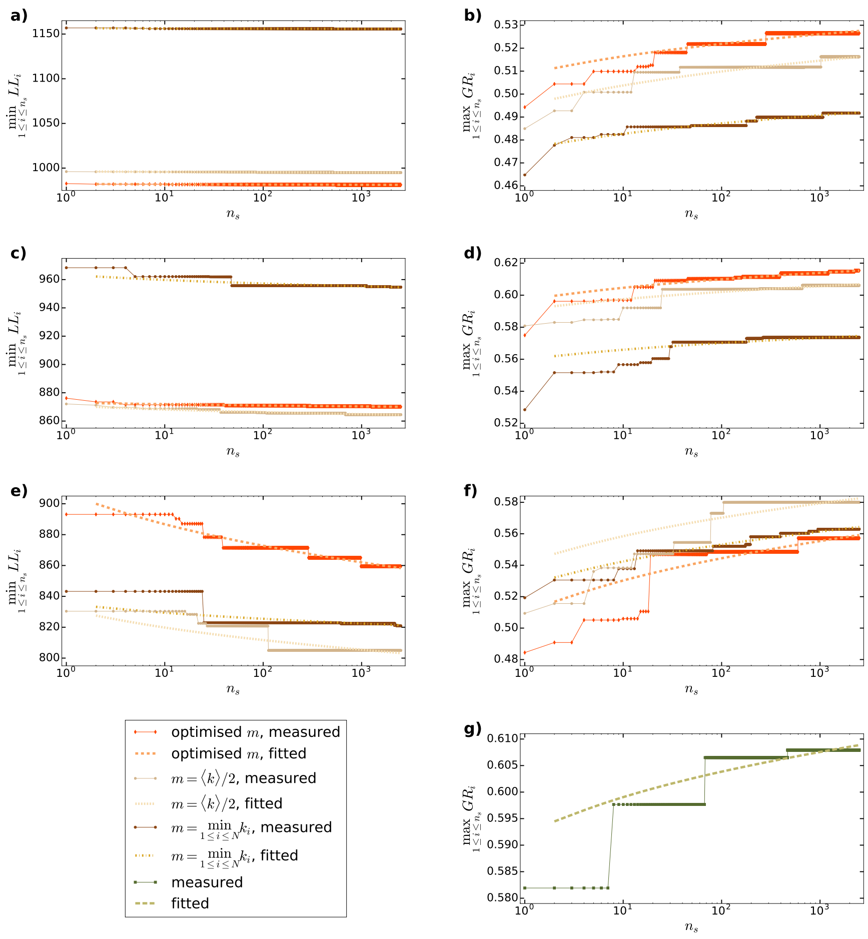

Figure S13. **The achieved best logarithmic loss (left) and greedy routing score (right) as a function of the number of repetitions of the embedding in the case of the network between books about U.S. politics.** Each row of the figure presents the results regarding one of the studied embedding algorithms: panels a) and b) refer to ncMCE, panels c) and d) refer to ncMCE with angular optimisation, panels e) and f) refer to HyperMap and panel g) refers to Mercator. Excepting Mercator, all the embedding methods were tested using 3 different parameter settings: either m was optimised together with β and T, or only β and T were optimised with m fixed to the half of average degree or the smallest occurring degree. The dashed curves were obtained by fitting the corresponding formulae in equations (S1a-S2) to the solid curves. The fitted coefficients and the quality of the fits are listed in Table S3.

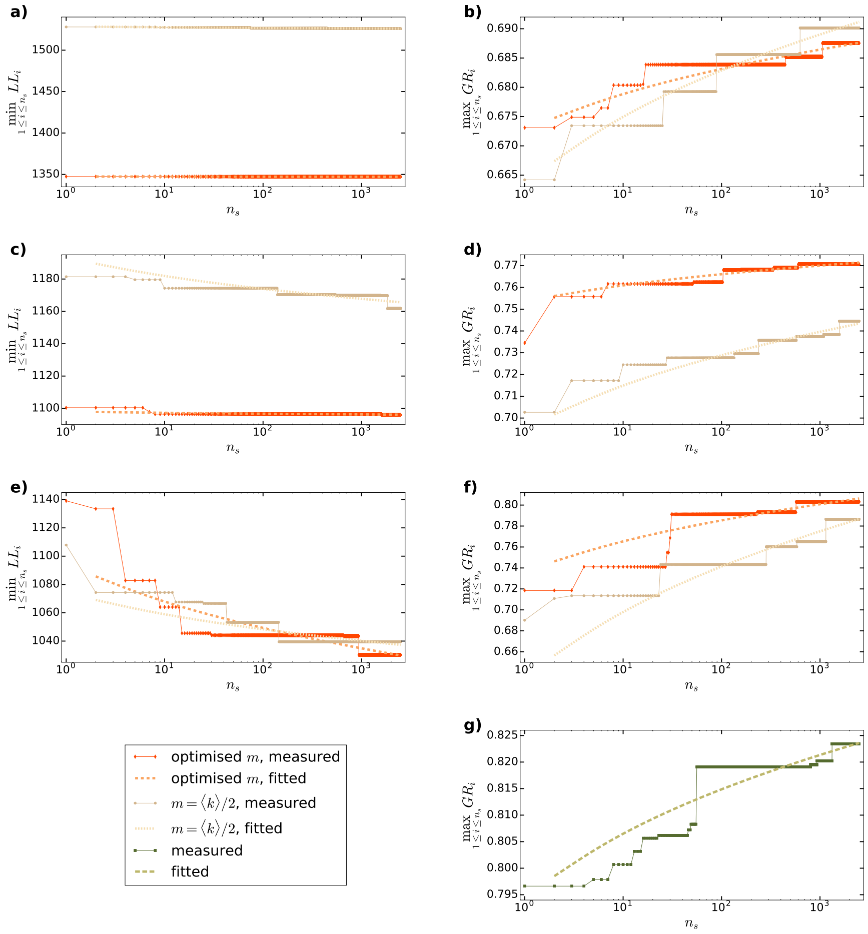

Figure S14. The achieved best logarithmic loss (left) and greedy routing score (right) as a function of the number of repetitions of the embedding in the case of the American College Football network. Each row of the figure presents the results regarding one of the studied embedding algorithms: panels a) and b) refer to ncMCE, panels c) and d) refer to ncMCE with angular optimisation, panels e) and f) refer to HyperMap and panel g) refers to Mercator. Excepting Mercator, all the embedding methods were tested using 2 different parameter settings: either m was optimised together with β and T, or only β and T were optimised with m fixed to the half of average degree. The dashed curves were obtained by fitting the corresponding formulae of equations (S1a-S2) to the solid curves. The fitted coefficients and the quality of the fits are listed in Table S4.

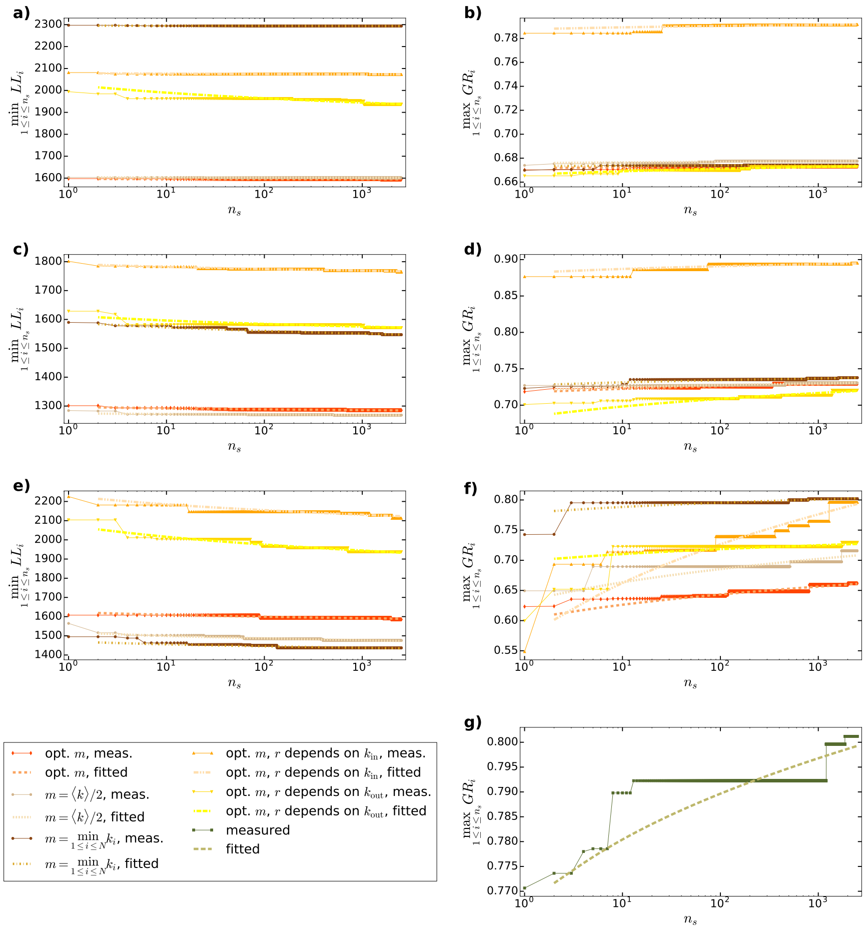

Figure S15. The achieved best logarithmic loss (left) and greedy routing score (right) as a function of the number of repetitions of the embedding in the case of the Cambrian food web from the Burgess Shale. Each row of the figure presents the results regarding one of the studied embedding algorithms: panels a) and b) refer to ncMCE, panels c) and d) refer to ncMCE with angular optimisation, panels e) and f) refer to HyperMap and panel g) refers to Mercator. Excepting Mercator, all the embedding methods were tested using 5 different parameter settings: either m was optimised together with β and T using a radial order determined by the total, the in- or the out-degree of the nodes, or only β and T were optimised with m fixed to the half of average degree or the smallest occurring degree. The dashed curves were obtained by fitting the corresponding formulae of equations (S1a-S2) to the solid curves. The fitted coefficients and the quality of the fits are listed in Table S5.

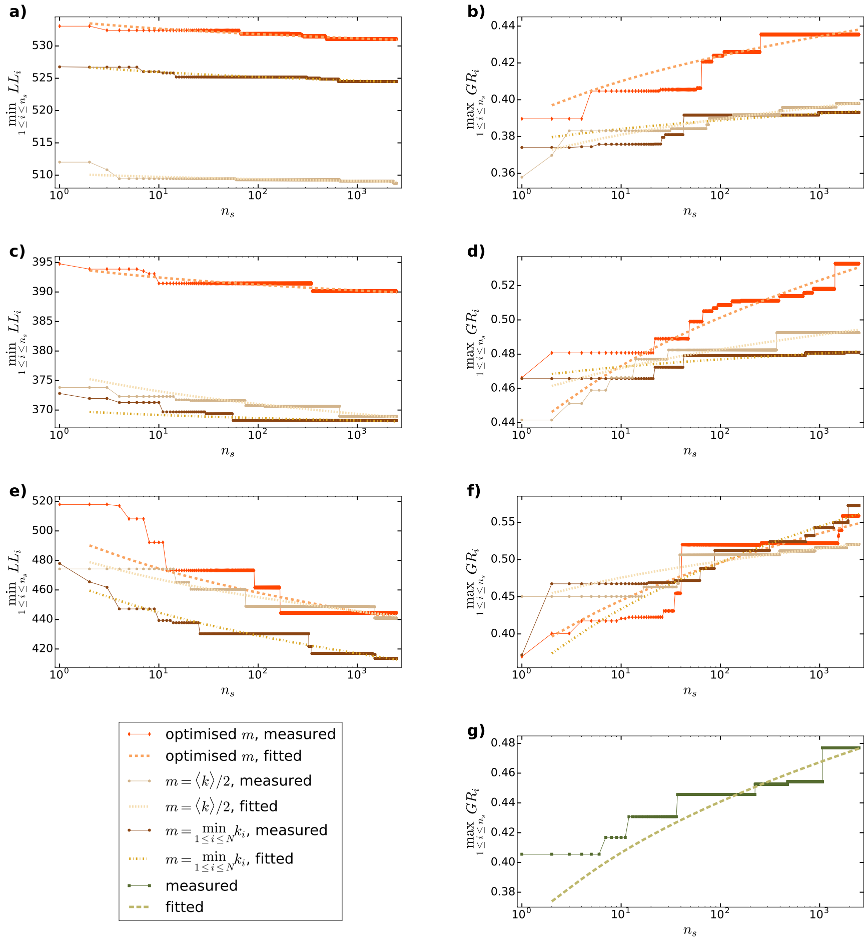

Figure S16. The achieved best logarithmic loss (left) and greedy routing score (right) as a function of the number of repetitions of the embedding in the case of the protein interaction network from the PDZBase database. Each row of the figure presents the results regarding one of the studied embedding algorithms: panels a) and b) refer to ncMCE, panels c) and d) refer to ncMCE with angular optimisation, panels e) and f) refer to HyperMap and panel g) refers to Mercator. Excepting Mercator, all the embedding methods were tested using 3 different parameter settings: either m was optimised together with β and T, or only β and T were optimised with m fixed to the half of average degree or the smallest occurring degree. The dashed curves were obtained by fitting the corresponding formulae of equations (S1a-S2) to the solid curves. The fitted coefficients and the quality of the fits are listed in Table S6.

Table S3. The results of fitting the formulae in equations (S1a-S2) to the curves plotted in Fig. S13 showing the achieved best quality scores as a function of the number of embeddings carried out in the case of the network between books about U.S. politics. The fitted coefficients are the mean (μ_{LL} and μ_{GR}) and the standard deviation (σ_{LL} and σ_{GR}) characterising the peak on that side of the quality score's observed distribution which corresponds to the best results. The quality of the fits is characterised by the coefficient of determination (R^2_{LL} and R^2_{GR}), which is a sufficiently high value in most of the cases. For the three embedding methods that were tried out with various parametrisations, the results regarding the eventually (after 2500 repetitions) best parameter setting are written in bold.

Embedding method	μ_{LL}	σ_{LL}	R^2_{LL}	μ_{GR}	σ_{GR}	R^2_{GR}
ncMCE, optimised m	**982.38**	**0.321**	**0.78**	**0.51**	**0.006**	**0.72**
ncMCE, $m = \langle k \rangle /2$ and $L = 0$	996.44	0.425	0.89	0.49	0.007	0.67
ncMCE, $m = \min_{1 \leq i \leq N} k_i$ and $0 < L = \langle k \rangle /2 - m$	1156.67	0.264	0.72	0.48	0.005	0.88
ncMCE with ang. opt., optimised m	873.37	0.917	0.83	**0.60**	**0.006**	**0.88**
ncMCE with ang. opt., $m = \langle k \rangle /2$ and $L = 0$	**871.51**	**2.045**	**0.82**	0.59	0.005	0.67
ncMCE with ang. opt., $m = \min_{1 \leq i \leq N} k_i$ and $0 < L = \langle k \rangle /2 - m$	964.21	2.734	0.62	0.56	0.005	0.54
HyperMap, optimised m	911.24	15.031	0.89	0.51	0.015	0.71
HyperMap, $m = \langle k \rangle /2$ and $L = 0$	**834.06**	**8.748**	**0.53**	**0.54**	**0.013**	**0.51**
HyperMap, $m = \min_{1 \leq i \leq N} k_i$ and $0 < L = \langle k \rangle /2 - m$	836.44	4.295	0.40	0.52	0.012	0.91
Mercator	–	–	–	0.59	0.005	0.61

Table S4. The results of fitting the formulae in equations (S1a-S2) to the curves plotted in Fig. S14 showing the achieved best quality scores as a function of the number of embeddings carried out in the case of the American College Football network. The fitted coefficients are the mean (μ_{LL} and μ_{GR}) and the standard deviation (σ_{LL} and σ_{GR}) characterising the peak on that side of the quality score's observed distribution which corresponds to the best results. The quality of the fits is characterised by the coefficient of determination (R^2_{LL} and R^2_{GR}), which is a sufficiently high value in most of the cases. For the three embedding methods that were tried out with various parametrisations, the results regarding the eventually (after 2500 repetitions) best parameter setting are written in bold.

Embedding method	μ_{LL}	σ_{LL}	R^2_{LL}	μ_{GR}	σ_{GR}	R^2_{GR}
ncMCE, optimised m	**1347.5**	**0.10**	**0.88**	0.67	0.005	0.78
ncMCE, $m = \langle k \rangle /2$ and $L = 0$	1529.0	0.85	0.86	**0.66**	**0.009**	**0.84**
ncMCE with ang. opt., optimised m	**1098.3**	**0.61**	**0.40**	**0.75**	**0.006**	**0.82**
ncMCE with ang. opt., $m = \langle k \rangle /2$ and $L = 0$	1195.9	8.60	0.46	0.69	0.015	0.83
HyperMap, optimised m	**1100.8**	**20.14**	**0.66**	**0.73**	**0.022**	**0.72**
HyperMap, $m = \langle k \rangle /2$ and $L = 0$	1077.6	11.44	0.59	0.62	0.047	0.82
Mercator	–	–	–	0.79	0.009	0.77

embedding method.

References

1. Muscoloni, A., Thomas, J. M., Ciucci, S., Bianconi, G. & Cannistraci, C. V. Machine learning meets complex networks via coalescent embedding in the hyperbolic space. *Nat. Commun.* **8**, 1615, DOI: 10.1038/s41467-017-01825-5 (2017).

2. Papadopoulos, F., Psomas, C. & Krioukov, D. Network mapping by replaying hyperbolic growth. *IEEE/ACM Transactions on Netw.* **23**, 198–211, DOI: 10.1109/TNET.2013.2294052 (2015).

3. García-Pérez, G., Allard, A., Serrano, M. Á. & Boguñá, M. Mercator: uncovering faithful hyperbolic embeddings of complex networks. *New J. Phys.* **21**, 123033, DOI: 10.1088/1367-2630/ab57d2 (2019).

4. Cramer, H. *Mathematical Methods of Statistics.*, chap. 28.6, 390 (Princeton University Press, 1999).

5. The Pierre Auger collaboration network was downloaded from the comune lab website: https://comunelab.fbk.eu/data.php. (Accessed: 24/02/2020).

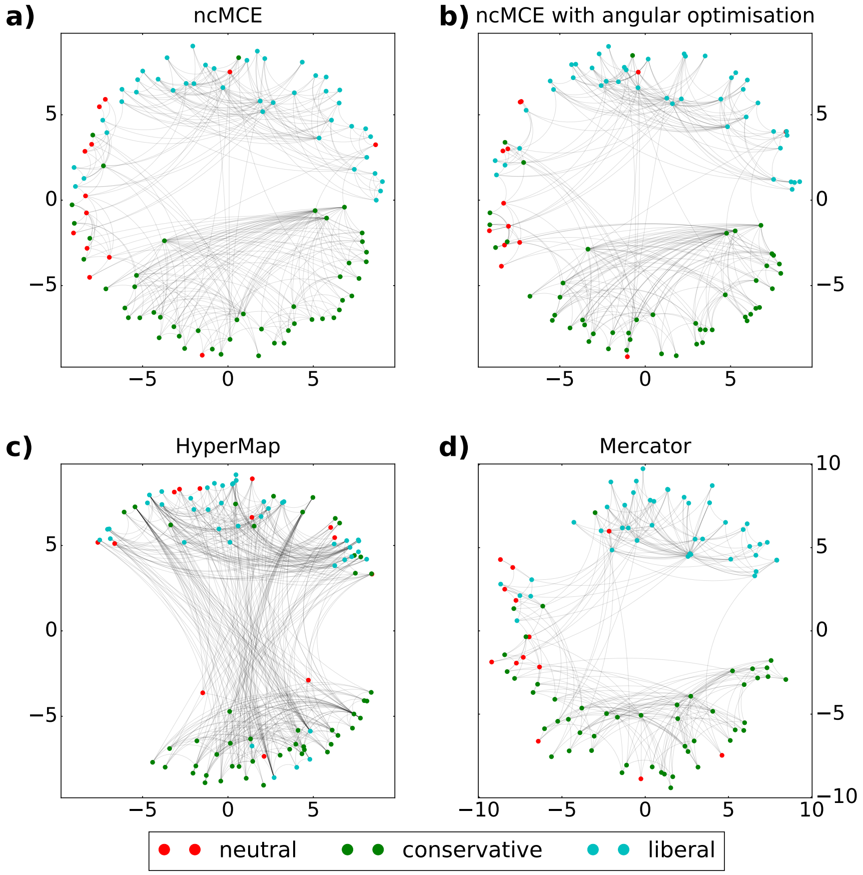

Figure S17. The layouts of the network between books about U.S. politics on the native hyperbolic disk that reached the highest greedy routing scores. a) The layout based on the coordinates resulted from the original ncMCE method. b) The layout according to the coordinates obtained with our approach, optimising the results of ncMCE. c) The hyperbolic layout obtained with HyperMap. d) The embedding according to Mercator.

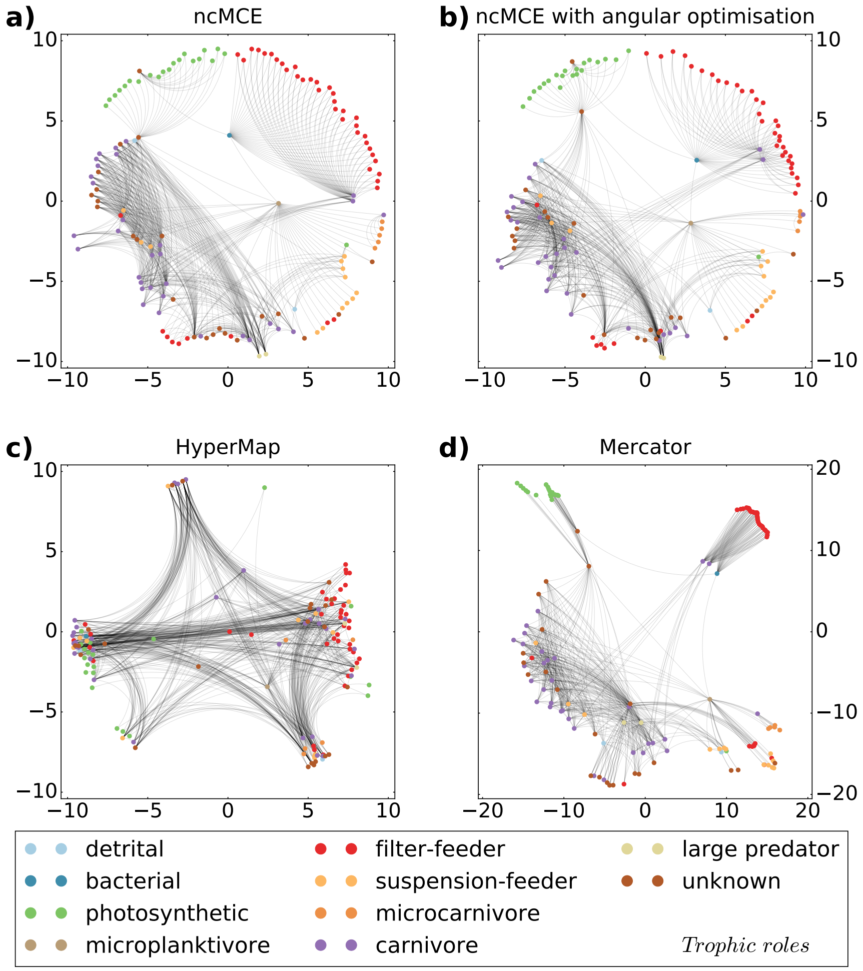

Figure S18. The layouts of the Cambrian food web on the native hyperbolic disk that reached the highest greedy routing scores. a) The layout based on the coordinates resulted from the original ncMCE method. b) The layout according to the coordinates obtained with our approach, optimising the results of ncMCE. c) The hyperbolic layout obtained with HyperMap. d) The embedding according to Mercator.

Table S5. The results of fitting the formulae in equations (S1a-S2) to the curves plotted in Fig. S15 showing the achieved best quality scores as a function of the number of embeddings carried out in the case of the Cambrian food web from the Burgess Shale. The fitted coefficients are the mean (μ_{LL} and μ_{GR}) and the standard deviation (σ_{LL} and σ_{GR}) characterising the peak on that side of the quality score's observed distribution which corresponds to the best results. The quality of the fits is characterised by the coefficient of determination (R^2_{LL} and R^2_{GR}), which is a sufficiently high value in most of the cases. For the three embedding methods that were tried out with various parametrisations, the results regarding the eventually (after 2500 repetitions) best parameter setting are written in bold.

Embedding method	μ_{LL}	σ_{LL}	R^2_{LL}	μ_{GR}	σ_{GR}	R^2_{GR}
ncMCE, optimised m	**1596.8**	**0.59**	**0.64**	0.672	0.0004	0.43
ncMCE, $m = \langle k \rangle /2$ and $L = 0$	1603.8	0.63	0.79	0.676	0.0006	0.52
ncMCE, $m = \min_{1 \leq i \leq N} k_i$ and $0 < L = \langle k \rangle /2 - m$	2295.4	0.50	0.76	0.673	0.0003	0.30
ncMCE, optimised m, radial order according to k_{in}	2079.0	1.58	0.48	**0.787**	**0.0012**	**0.37**
ncMCE, optimised m, radial order according to k_{out}	2034.9	27.64	0.67	0.665	0.0022	0.65
ncMCE with ang. opt., optimised m	1298.3	3.80	0.82	0.716	0.0039	0.71
ncMCE with ang. opt., $m = \langle k \rangle /2$ and $L = 0$	**1275.3**	**1.93**	**0.71**	0.719	0.0033	0.63
ncMCE with ang. opt., $m = \min_{1 \leq i \leq N} k_i$ and $0 < L = \langle k \rangle /2 - m$	1593.7	13.14	0.82	0.726	0.0033	0.70
ncMCE with ang. opt., optimised m, radial order according to k_{in}	1794.7	7.81	0.80	**0.880**	**0.0041**	**0.53**
ncMCE with ang. opt., optimised m, radial order according to k_{out}	1617.2	12.85	0.57	0.680	0.0113	0.68
HyperMap, optimised m	1626.9	10.74	0.55	0.596	0.0183	0.80
HyperMap, $m = \langle k \rangle /2$ and $L = 0$	1522.1	13.82	0.79	0.625	0.0236	0.51
HyperMap, $m = \min_{1 \leq i \leq N} k_i$ and $0 < L = \langle k \rangle /2 - m$	**1473.5**	**10.99**	**0.62**	**0.776**	**0.0076**	**0.68**
HyperMap, optimised m, radial order according to k_{in}	2238.2	32.71	0.63	0.549	0.0693	0.79
HyperMap, optimised m, radial order according to k_{out}	2087.4	43.79	0.86	0.696	0.0089	0.33
Mercator	–	–	–	0.764	0.0100	0.53

Table S6. The results of fitting the formulae in equations (S1a-S2) to the curves plotted in Fig. S16 showing the achieved best quality scores as a function of the number of embeddings carried out in the case of the protein interaction network from the PDZBase database. The fitted coefficients are the mean (μ_{LL} and μ_{GR}) and the standard deviation (σ_{LL} and σ_{GR}) characterising the peak on that side of the quality score's observed distribution which corresponds to the best results. The quality of the fits is characterised by the coefficient of determination (R^2_{LL} and R^2_{GR}), which is a sufficiently high value in most of the cases. For the three embedding methods that were tried out with various parametrisations, the results regarding the eventually (after 2500 repetitions) best parameter setting are written in bold.

Embedding method	μ_{LL}	σ_{LL}	R^2_{LL}	μ_{GR}	σ_{GR}	R^2_{GR}
ncMCE, optimised m	534.17	0.916	0.82	**0.39**	**0.015**	**0.69**
ncMCE, $m = \langle k \rangle /2$ and $L = 0$	**510.35**	**0.378**	**0.57**	0.37	0.009	0.91
ncMCE, $m = \min_{1 \leq i \leq N} k_i$ and $0 < L = \langle k \rangle /2 - m$	527.28	0.812	0.81	0.38	0.005	0.59
ncMCE with ang. opt., optimised m	394.64	1.334	0.69	**0.42**	**0.031**	**0.78**
ncMCE with ang. opt., $m = \langle k \rangle /2$ and $L = 0$	376.99	2.335	0.76	0.45	0.012	0.74
ncMCE with ang. opt., $m = \min_{1 \leq i \leq N} k_i$ and $0 < L = \langle k \rangle /2 - m$	**370.11**	**0.574**	**0.46**	0.47	0.005	0.72
HyperMap, optimised m	503.44	17.709	0.62	0.36	0.055	0.60
HyperMap, $m = \langle k \rangle /2$ and $L = 0$	488.71	13.157	0.67	0.44	0.024	0.79
HyperMap, $m = \min_{1 \leq i \leq N} k_i$ and $0 < L = \langle k \rangle /2 - m$	**472.22**	**16.882**	**0.84**	**0.32**	**0.068**	**0.83**
Mercator	–	–	–	0.35	0.037	0.74

6. The network between books about u.s. politics was downloaded from https://www-personal.umich.edu/ mejn/netdata/polbooks.zip. (Accessed: 24/02/2020).

7. The american college football network was downloaded from https://www-personal.umich.edu/ mejn/netdata/football.zip. (Accessed: 24/02/2020).

8. Dunne, J. A., Williams, R. J., Martinez, N. D., Wood, R. A. & Erwin, D. H. Compilation and network analyses of cambrian food webs. *PLoS Biol.* **6**, e102, DOI: 10.1371/journal.pbio.0060102 (2008).
9. The network of pdz-domain-mediated protein–protein binding interactions was downloaded from https://konect.cc/networks/maayan-pdzbase/. (Accessed: 24/02/2020).
10. The network of hyperlinks between blogs on u.s. politics was downloaded from http://www-personal.umich.edu/ mejn/netdata/polblogs.zip. (Accessed: 24/09/2020).
